# Proximal procrastination of saccades: “paradoxical” delays to larger objects explained by a simple payoff-time heuristic

**DOI:** 10.1016/j.isci.2025.114426

**Published:** 2025-12-11

**Authors:** Mark R. Harwood

**Affiliations:** 1 Department of Biology, City College of New York, New York, NY 10031, USA; 2 Department of Psychology and Human Development, University of East London, London, UK

**Keywords:** Cognitive neuroscience, Life Sciences

## Abstract

Our most common decision is rife with procrastination: saccade latencies are typically 2-5x minimum sensorimotor pathway delays. Procrastination is explained as time to accumulate sensory evidence for target selection. Larger targets, or those nearer the fovea, have larger cortical representation and salience, and thus should elicit faster targeting. Paradoxically, the opposite is true. We present the largest parametric effect on saccade preparation timing in simple objects (>100 ms). Proximal or larger targets were systematically delayed, with relative eccentricity explaining 88% of latency variance. A payoff-time model pitting information gained from peripheral preview against that from foveation explained both object-sized and foveal microsaccade latency peaks. Free choices between proximal (high salience-by-proximity) and distal (high motor-priority) targets found priority dominating. Collectively, these results unite disparate findings in oculomotor literature around a proximal procrastination rule. The extraordinary structure and strength of the latency data, coupled with naturalistic saccades being object-targeted, imply wide scientific and, potentially, applied importance.

## Highlights

• Object-size sets “motor intensity” parallel to Pieron’s Law for saccade latencies
• Largest effect on saccade preparation decision: parametric proximal procrastination
• Foveal-Perifoveal Trade-off of information gain predicts latencies
• A “free choice” paradigm has choices obligatorily set by proximal procrastination

## Graphical abstract

**Figure:**
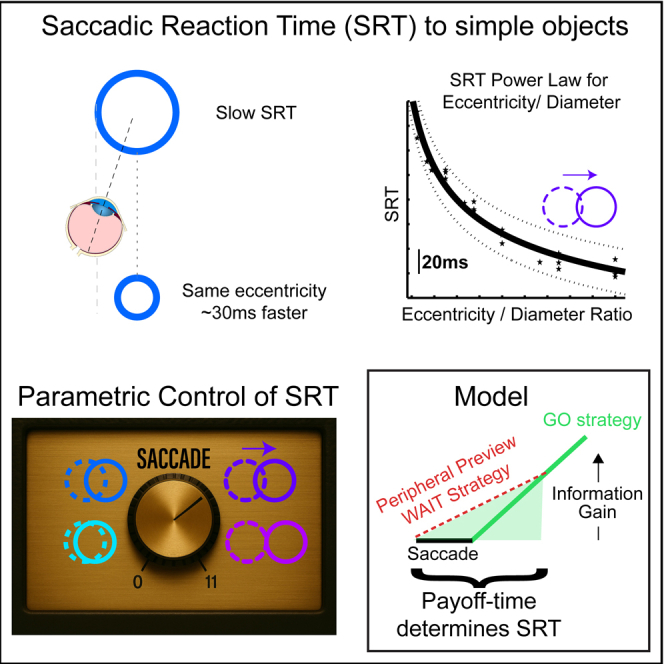

## Introduction

Unlike a camera, the human eye has an extremely non-uniform resolution across space. Within 25° eccentricity, 20/20 central vision falls below 20/200 (top line of an eye chart) used in legal definitions of blindness. Visual “clutter” in the periphery degrades processing even more strongly by crowding our already limited resolution there.^1^ Of course, we are largely unaware of this drastic loss of acuity vision because our eyes are almost constantly realigning the optimal foveal vision on the object of our immediate interest. We make these saccadic gaze shifts twice as often as we beat our hearts, and yet each one requires a decision both of where and when to move. Despite their frequency, saccade decisions are remarkably slow and variable even to single targets. Afferent and efferent pathway delays are as low as 60 ms,^2^^,^^3^^,^^4^ and yet the latency for a saccade to the step of a bright spot on a blank background is frequently 200–300 ms.^5^ Time is important to the brain, so why would this critical information gathering mechanism have such long and variable latencies?

A popular explanation is that the saccadic system deliberately procrastinates until the sensory evidence for the target location is sufficiently compelling.^6^^,^^7^ Because the saccade system is usually faced with a multiplicity of targets, it tends to delay saccades beyond their physiological minimum to ensure that an appropriate target is selected and to avoid excessive movements. Different target locations compete in a race for selection, accumulating sensory evidence sequentially until a criterion finishing line is reached, whereupon a saccade to that target is executed. This helpful cautionary restraint is difficult to disengage volitionally, operating even for single isolated targets,^6^^,^^8^ where there is no prima facie need for a decision beyond detecting target presence. Thus, in the saccade reaction time (SRT, or “latency”) literature, “target selection” is visual and has often been equated to the key decision stage; anything after is a “response selection” decision, or motor preparation planning. Various forms of sensory-weighting accumulator models exist, which all replicate reaction times well: linear,^9^ diffusion,^10^ and leaky integrator.^11^ Neural signals in the frontal^12^ and parietal^13^ cortices and superior colliculus^14^^,^^15^ confirm these models via accumulator rates and variability that match SRT distributions.

More broadly, neurophysiologically supported accumulator models underpin perceptual decision making tasks,^16^^,^^17^^,^^18^^,^^19^^,^^20^^,^^21^ and can explain Pieron’s law,^22^^,^^23^^,^^24^ which states that mean response times decrease as a power function of stimulus intensity in multiple sensory modalities (vision,^25^ audition,^26^ olfaction,^27^ gustation^28^). Integrating stimulus intensities of various features, such as luminance and color contrast, into a measure of “salience” has proved hugely influential in visual search in determining the likelihood of one region being selected by attention or saccadic eye movements.^29^ The above approaches have relied on the feedforward accumulation of sensory inputs, however the response selection is also affected by the behavioral goal. Behavioral tasks with fixed sensory inputs can modulate accumulation rates.^30^^,^^31^^,^^32^^,^^33^^,^^34^ Perceptual decisions are modulated by motor urgency,^35^^,^^36^^,^^37^ and top-down prioritization of response modulates saliency in visual search.^38^

Critically, sensory-accumulator SRT models are challenged by evidence that most of the procrastination and variability arises in motor preparation after target selection. Primate physiology^39^ and the human “temporal impulse function”^40^ have shown that SRT only correlates with the delay after sensory information has been used to select the target for the saccade. Splitting accumulator models into initial sensory processing and a later “gated” motor preparation accumulation^41^^,^^42^^,^^43^ describes the behavioral and physiological data better, but still does not explain why motor preparation is so long and variable.

Motor preparation remains enigmatic, in part due to limited experimental manipulation tools. Unlike sensory processing, whose input can be continuously varied by the experimenter, motor preparation or priority modulations have typically been discrete (e.g., varying the number of choices,^44^ or binary task differences^38^). Previously, we found a promising handle on motor preparation dependent on the spatial scale of attention.^32^^,^^45^ Instructing participants to attend to one of the two concentric segmented rings that rotated (changing segment number unpredictably) allowed control of attention scale and titrated performance, while measuring SRT to steps of the compound stimuli. Although the sensory stimulus was identical, SRTs were doubled when attending to the large ring.^45^ We found that larger and nearer targets had longer SRTs, with the ratio of eccentricity-to-attention-scale modulating the accumulating decision signal rate as inferred from modeling of SRT distributions.^32^ There was no kinematic speed-accuracy trade-off and only the inverted trade-off of initiation time (SRT) being slower to larger targets. Neither divided attention nor saccade amplitude could explain the data. We found neither interaction between contrast and attention scale in our SRT, nor differences in target-step detection reaction times between scales. Collectively, we concluded that the eccentricity-to-attention scale modulated the motor preparation urgency (response priority) by scaling the accumulation rate of the sensory evidence.

The results from those attention-task experiments were “paradoxical” from sensory-led decision-making and simple speed/accuracy trade-off perspectives, because larger targets had more delayed responses. They also clash with findings of limited effects of combining eccentricity and spatial scale in classic reflexive saccade paradigms.^46^^,^^47^^,^^48^^,^^49^^,^^50^ Although our attention-scale SRT data have been supported in some data from reflexive paradigms,^51^ those experiments had a limited range of conditions. Hence, a key aim of the current study was to firmly establish whether relative eccentricity-to-scale has a *parametric* effect on saccade motor preparation to simple objects of different sizes. This is particularly important given that naturalistic saccades are made to objects of varying sizes, and we propose that the largest determinant of SRT may be object eccentricity-to-size ratio.

Here, we first demonstrate striking structure in SRTs driven by a target’s relative eccentricity-to-size, which is the largest discovered parametric effect on saccade motor preparation (∼100 ms). Then, we develop an explicit cost/benefit payoff-time model to explain this structure based upon the key intrinsic function of saccades: resolution benefits of foveation. We apply the same model to explain the “central peak” of SRT, where SRT increases systematically as eccentricity decreases within the fovea^52^^,^^53^^,^^54^^,^^55^^,^^56^^,^^57^; this demonstrates that longer reactions to targets stepping to very small eccentricities (<2°) are systematically delayed rather than forming a “dead zone” in which one simply does not bother making movements. Finally, we apply the same model in a volitional paradigm, predicting free choices between pairs of targets, which gives opposite predictions to salience models, and raises the possibility that this may be a key unexplored feature of visual search more broadly.

## Results

To demonstrate parametric control over saccade preparation timing and reveal the structure in SRT distributions, in Experiment 1, we simply had participants follow single ring targets stepping to various eccentricities.

### Object scale structures saccade reaction time

**Figure 1:**
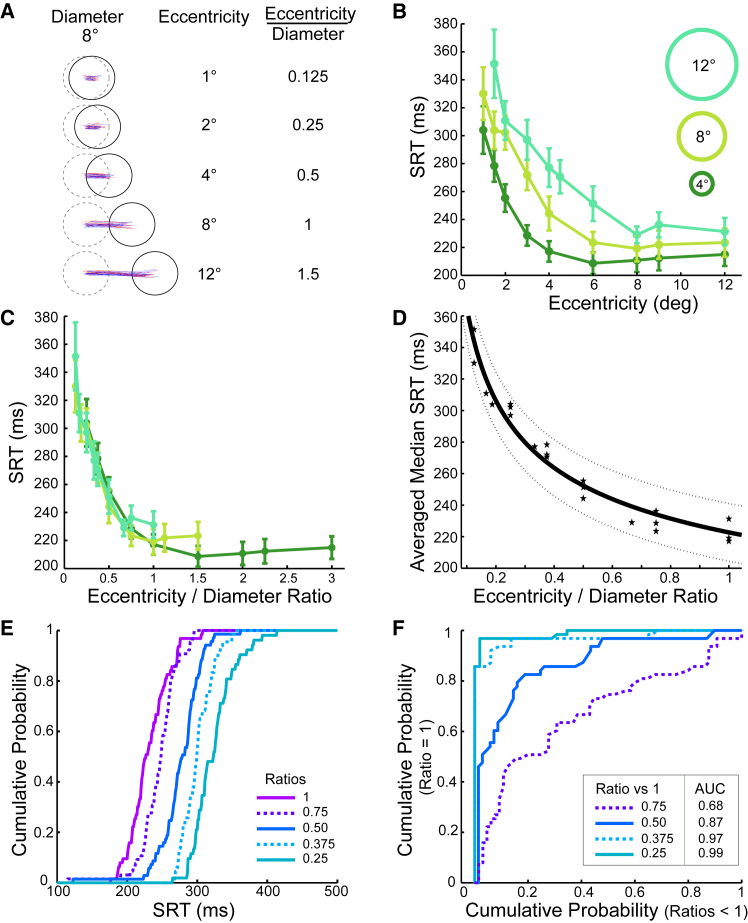
Saccade reaction times to steps of single ring targets (Experiment 1) (A) Example set of conditions showing saccade trajectories for one participant (red: rightward; blue: leftward but reflected horizontally). Dashed circle is the ring starting at screen center (preceding fixation spot not shown) before stepping right or left. Rings always stepped from the screen center by ±0.5-12°. Non-ageing foreperiods were used (see STAR Methods). (B) Median SRT vs. eccentricity for 4, 8, 12° ring diameters plotted against eccentricity (colors are only labels, all rings were red). Median SRT in each condition was averaged across the 6 participants (see Figure S1 for individual data). Error bars denote SEM. A linear mixed model showed significant effects of eccentricity (F(9,130) = 50.4, *p* < .001), diameter (F(2,130) = 52.9, *p* < .001), and an interaction that reduced the effect of eccentricity as ring diameter increased (F(15,130) = 4.9, *p* = .029). (C) Replott of the Median SRT against the unitless eccentricity/ring diameter ratio, which reduced SRT (F(14,130) = 33.9, *p* < .001) without interaction or additional effects of diameter (*p* > .05). (D) Power law fitting of the non-plateau region (eccentricity/diameter ratios up to 1) with 95% intervals (dotted curves); data points (star symbols) are collapsed across participants, not ring sizes. (E) SRT cumulative distribution functions for one subject showing approximately parallel shifted distributions with almost complete separation between ratios = 1 (purple) and 4 ratios <1 (shades of purple-to-blue: 0.75 dotted; 0.5; 0.375, dotted; 0.25). (F) Receiver-operating characteristic curves from plotting the Ratio = 1 condition (purple) distribution in E to the other 4 ratio conditions showing 68–99% area-under-curves. (See also Figure S2 for control experiments showing no effect on manual reaction time, effects of fixation offset conditions, and no difference between rings and discs; Figure S3 for single ring data in the 12 participants of Experiment 3; Figure S4 for equivalent monkey SRT data.).

After fixating a small (0.3°) spot at the screen center, six participants fixated a ring (4, 8, or 12° diameter) at the same location that then stepped randomly left or right from the screen center by 1, 1.5, 2, 3, 4, 4.5, 6, 8, 9, or 12°. All but one were naive participants, and they were simply instructed to follow the ring as quickly as possible whenever it stepped peripherally (without explicit instructions to target the center or ring edges). An example set of conditions and trajectories are shown in Figure 1A. Only trials containing saccades in the correct direction within 80–600 ms of the target step were considered for analysis, with online eye velocity and a horizontal fixation window ( ±0.5°) used to abort trials with blinks or movements before 80 ms. These naive participants had fewer valid trials (79%) compared to the similar ring paradigm conditions in Experiment 3 using experienced participants (93% valid trials). There were 27 combinations (3 ring diameters x 9 eccentricities) of eccentricity and ring diameter designed to generate 15 eccentricity/diameter ratios of 0.125–3.

SRT increased strongly with ring diameter and decreased strongly with the eccentricity of the peripheral target. All participants displayed a similar pattern, and the statistics are presented below after describing the key consistent features in the data. The average across participants is shown for the median SRT in each condition (Figure 1B) (for individual data, see Figure S1). Larger rings elicited much longer SRTs for a given eccentricity; nearer eccentricity targets also evoked saccades with much longer SRT. When these two parameters of target step amplitude and width were combined in the Eccentricity/Diameter Ratio (Figure 1C), the curves for the different rings tended to collapse onto a single function of SRT, such that SRT was strongly predicted by this simple Ratio parameter. Small deviations (SRT above the collapsed curve) can be seen from 2 of the 27 conditions tested, arising from the largest eccentricity tested here, 12°, for the 8 and 12° ring diameters. Those are consistent with the known SRT increase outside of the macula (e.g., Becker,^5^ 1989). The second key feature was SRT hitting a minimum plateau for eccentricity/diameter ratios above one. Note, essentially all past reflexive saccade paradigms fall in this plateau region (e.g., a typical saccade target of 0.3° diameter stepping by 9° would be ratio = 30). Again, there is a small trend toward the SRT increasing in the ratios >1 plateau region as the eccentricity increases beyond 5–10°, consistent with standard SRT literature.

To statistically test the ability of eccentricity/diameter ratio to parametrize SRT, log-log regressions were fit to the power law relationship seen in the SRT-changing region up to a ratio of 1. Regressions on the data averaged across participants (Figure 1D) showed a power law: *SRT= a∗(eccentricity/diameter)*^*b*^ with significant slope *b* = - 0.22 (*t*(18) = 24.5; *p* < 0.001) and constant *a* = 218 ms. The eccentricity/diameter ratio accounted for 97% of the group median SRT variance (*r*^2^ = 0.97, F(1, 18) = 600.1, *p* < 0.001), suggesting a lawful effect of object size and eccentricity on SRT. Individual fits for each participant all gave significant power law slopes between median SRT and eccentricity/diameter (all *p* < 0.001), with strong effect sizes accounting for the majority of variance in individual SRT medians (*r*^2^ = 80–97%; group average = 88%). Indeed, individual trial data for each participant gave strong fits on a saccade-by-saccade basis (slopes all *p* < 0.001); effect sizes ranged from *r*^2^ = 0.15 (F(1,325) = 58.4, *p* < 0.001) to *r*^2^ = 0.53 (F(1,410) = 455.3, *p* < 0.001). The average ratio effect size explained 35% of individual saccade-to-saccade SRT variance. Adding absolute saccade amplitude to the regression did not change the model (R^2^-change = 0.00, *p* > 0.05) with a negligible regression slope (B = −0.01, *p* > 0.05).

We argue that the eccentricity/diameter parameter reflects a saccade motor preparation intensity rule. Control experiments here (and previously^32^) showed that manual reaction times were not influenced by eccentricity/diameter conditions, indicating that all conditions were equally detectable (Figure S2A). Differences in the detectability of stimulus steps are not thought to be important in stimuli well above threshold contrast,^41^ and could not explain the magnitude of the suprathreshold SRT differences in Figure 1. Because detectability and contrast do not change the effect, and other visual or attentional factors have been excluded,^32^ we interpret this as a saccade motor preparation intensity rule.

Motor preparation is often synonymous with motor planning; could increased difficulty in targeting the center of larger objects cause the increased SRT of Figure 1? If the explanation of the observed doubling in SRT were true, one might also expect large increases in SRT with increasing eccentricity, because visual and motor precision decreases in the periphery. Instead, SRT falls dramatically with eccentricity over the range where object size is important. Moreover, saccade precision to spatially extended targets has been shown to be remarkably precise with very little impact on SRT,^48^ so a simple target planning delay is unlikely to explain the large, systematic effect in Figure 1.

To further demonstrate the primacy and reproducibility of the eccentricity/diameter ratio in explaining our SRT data, repeated-measures ANOVA was run across the 18 participants in comparable conditions pooled across Experiments 1 and 3. Twelve participants in the control condition of Experiment 3 followed single ring targets of 2, 4, and 8°, and demonstrated the same pattern as in Experiment 1 (Figure S3). Over the 5 overlapping ratio conditions, there was a strong main effect of eccentricity/diameter ratio (F(1.07, 11.7) = 36.8, *p* < 0.001, η_p_^2^ = 0.77), but no other effect of ring size or interaction between ratio and ring size (*p* > 0.05). We have recorded dozens of other participants in earlier experimental versions with fewer ratio conditions (unpublished), and the pattern is universal with no outliers. In sum, when there is no overlap in stimulus locus between the initial and final target location (ratios >1), latencies are short, and progressively increase as the pre/post overlap increase (ratios <1).

Although the key result is that this eccentricity/diameter manipulation allows parametric control over SRT, the 122 ms magnitude of the spatial scale effect (difference between SRT at the lowest 0.125 ratio and ratio = 1 in Figure 1D) is also helpfully larger than the paradigms that have dominated the SRT literature over the last 50 years. The ratio had such a strong effect on SRT for ratios <1 that comparing cumulative distribution functions (CDFs) of low and high ratios shows essentially no overlap (Figure 1E, representative naive participant). Plotting CDF for the condition of ratio = 1 against lower ratios (Figure 1F), shows area-under-curve classification accuracies of up to 99%. Remarkably, this indicates that one can not only predict median SRT from target size, but given two targets stepping by ratios of 1 or 0.125, one could predict with 99% accuracy which one this participant was reacting to from a single reaction time.

Classic saccade motor preparation and attentional fixation disengagement paradigms are superimposed on the spatial scale effect. Having a ring extinguish 200 ms before appearing peripherally (Figure S2B, Gap), or having the initial ring remain on, overlapping with a new target (Figure S2B, Overlap) simply shifted the SRT-Ratio function by ∼20–40 ms in each direction (comparable magnitudes to typical spot Gap/Overlap effects^58^). Unlike the rings in Figure S2B, classic Gap/Overlap paradigms have a target over the central fovea, which have been thought to stimulate “fixation neurons” in the foveal region of the superior colliculus,^59^ delaying saccades. To minimize the argument that a low-level fixation neuron mechanism was responsible for the scale effect, we used rings throughout this study to avoid the direct visual stimulation of the central rostral collicular. However, to further demonstrate that the foveal fixation condition does not cause the Figure 1 changes in SRT, solid disc stimuli were interleaved with ring stimuli in a normal reflexive paradigm (no gap or overlap). There were no significant differences between solid discs and rings (Figure S2C; *p* > 0.05), demonstrating that the spatial scale effect is object-based rather than visual-energy-based.

The spatial scale effect is present in naive monkeys. The behavioral and neural correlates of Figure 1 are the subject of a companion article (Caziot et al., 2025).^60^ However, to demonstrate the wider applicability and obligatory nature of the phenomenon here, we show the first session in the first monkey with extended spatial scale targets (Figure S4). The monkey had previously undergone standard initial training to fixate on small targets. The data were noisier than the same task in an instructed human, but the pattern is strikingly similar.

If, as we argue, spatial scale drives the largest parametric effect reported on saccade motor preparation, how has such a simple, yet strong, scale effect remained previously unappreciated? In order to be sure of good fixation, small targets are frequently used (≤1°) in saccade research, meaning that any eccentricity >1° would be in the short-latency plateau region of Figure 1C (eccentricity/diameter ratios ≥1).^61^^,^^62^ Previous studies with extended targets have also mostly used the equivalent of Step/diameter ratios ≫ 1 (i.e., in the plateau region)^48^^,^^49^^,^^50^ and partly conflated eccentricity and size effects.^46^^,^^47^ Conversely, it has long been known that target eccentricities <1° give rise to a “central peak” in SRT, which has been shown to be of motor preparation not sensory origin.^52^^,^^53^^,^^54^ Until recently,^56^^,^^57^ the central SRT peak has received scant attention by others because it only applies within the foveola (<0.75° eccentricity). Our finding that a region of steep increase in SRT extends to much larger eccentricities and depends critically on object width suggests a fundamental constraint on saccadic decision making. This size-latency effect greatly increases the importance of understanding the central peak in SRTs. We develop an explanatory model below.

### Modeling the saccade decision signal

**Figure 2:**
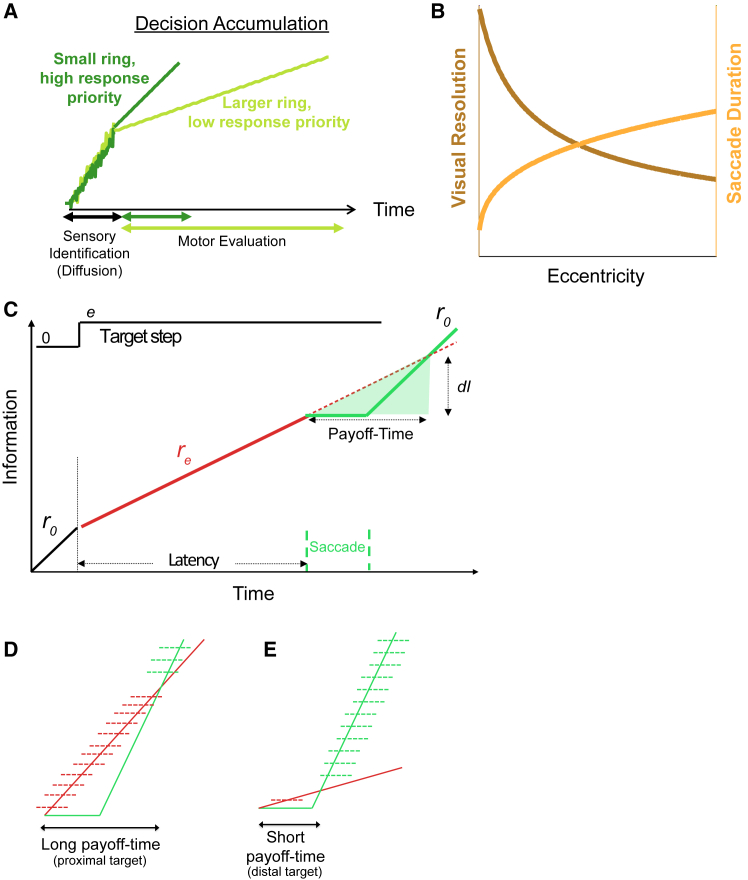
Saccade payoff-time decision model framework (A) Dual-stage mechanistic model in which high contrast single targets on blank backgrounds are all readily detected in a minimum sensory processing delay. A diffusion process is assumed for stage 1 that is size-invariant at our contrasts. Stage 2 sees motor preparation signals rise more slowly for larger targets. (B) Visual resolution falls and saccade durations rise as a function of eccentricity. By “resolution” we refer to reciprocal sampling spacing throughout, rather than sampling spacing (“minimum angle of resolution”) that increases quasi-linearly with eccentricity. (C) Information accumulation of the target (*r*_*0*_) falls when it steps away from the fovea (*r*_*e*_), while it is peripherally previewed and the decision of whether it is worth refoveating it is made. We assume no information gain during the saccade (flat portion), and a return to foveal rate thereafter. “Payoff-time” is the time after a saccade that the information lost during the saccade by the movement choice catches up with the remaining fixated choice. (D and E) Proximal targets lose less information during peripheral preview because the peripheral preview information gain (solid red line) is steeper. Thus, proximal targets have longer payoff-times for the movement choice (green) compared to distal targets. Horizontal dashed lines indicate hypothetical information gain goals, with colors showing which bounds are achieved first by fixation (red) versus saccade (green) response-mode choices. These bounds are simply to visualize how the probability ratio of achieving an uncertain information goal via fixation as opposed to saccade choice is higher for proximal than distal targets, and to show that this is proportional to payoff-time. In this scheme, longer payoff-times should have lower saccade-response priority, implemented via a lower slope in the second stage accumulator of Figure 2A.

Fitting SRT distributions to single linear accumulator^32^ or diffusion models^60^ shows that decision accumulator rate increases with eccentricity/diameter ratio (Figure S5; also see STAR Methods). Current models split accumulation into dual processes^8^^,^^33^ of visual selection and response selection components. Although contrast can delay SRT considerably,^63^^,^^64^ behavioral evidence in humans^40^^,^^41^^,^^65^ and neurophysiological evidence in monkeys^39^^,^^42^^,^^66^ show that suprathreshold contrast targets are visually selected in about 80 ms. Larger targets could only be expected to potentially reduce this temporal window of selection marginally (a ceiling effect on the diffusion rate), and visual transients have been reported^67^ to be insensitive to target size at contrasts above 20%. Moreover, previously we showed contrast minimally modulates the effect of spatial scale of attention on SRT over a suprathreshold range 20–80% contrast.^32^ Thus, in opposition to perceptual decision-making models that fix the motor planning time into the “non-decision time,” here we fix the visual selection time at 80 ms and have the whole variability in time to decision in the motor response part, with the spatial scale affecting only the response selection (Figure 2A).

In addition to relying on the existing diverse literature above to infer an approximately 80 ms boundary between visual selection and response selection decisions, we note that this dichotomy is consistent with our own experiments to dissect the delay, which are the subject of a forthcoming article. That work follows an equivalent methodology to the “temporal impulse function” experiments of Ludwig and colleagues (2005), but with the added factor of object size, and gave similar findings of an 80–100 ms visual selection window, before an accumulating motor preparation stage that is dependent on object size.

This dual stage model matched our cost/benefit argument^32^^,^^68^ for the spatial scale effect in terms of modulating only the response selection based upon an evaluation of the cost/benefit of a particular movement. The specific cost/benefit argument is developed in the next section, but the conceptual argument is that because larger targets are seen better at a given eccentricity than smaller targets, saccades to larger targets result in less foveation benefit relative to small targets, while carrying the same amplitude-dependent costs. The resultant cost/benefit ratio is lower for movements to larger targets. Response selections are set with different priorities, resulting in different accumulation rates of motor preparation. Instead of sensory accumulation-weighting favoring larger, nearer targets in a purely feedforward fashion, motor-waiting accumulation does the opposite, waiting longer for large nearby targets while prioritizing smaller, less salient targets based on the expected sensorimotor consequences of the response choice.

Saccades bring both sensorimotor costs and benefits. The visual resolution benefits of foveating a peripheral target are part-countered by costs in time, vision, and opportunity in doing so, given the large refractory period between movements.^69^ Saccade durations typically range from 20 to 80 ms, and refractory periods are ∼200 ms. The visual costs from saccadic suppression during the movement^70^ and misperceptions just before and after the saccade^71^^,^^72^ extend costs beyond the duration in-flight. Additional time is also often required in future corrective movements.^73^ There is also a 60 ms commitment “dead time”^74^ during which the system is committed to its movement choice. Thus, there are opportunity costs in moving rapidly to a “bad target” when a higher priority one may intervene during a period of commitment to the low priority target.

To extend the mechanistic model of Figure 2A into an explanatory model predicting prioritization rates, we first consider a simpler situation: reacting to point targets moving within the fovea (the SRT “central peak”), and compute costs and benefits of movement in a common currency, time.

### Payoff-time model

Visual resolution falls rapidly even within the central 4° of the retina,^57^^,^^75^ while saccade duration increases in a power function for small saccades^76^ before becoming quasi-linear for saccades to larger eccentricities (Figure 2B). Because of more photoreceptors, information about a parameter of interest will increase most sharply at the maximal sampling of the cone mosaic; under standard sampling assumptions, variance in the parameter estimate will be inversely proportional to samples (in time), and thus precision will increase linearly at rate, *r*_*0*_ (Figure 2C), and the rate-of-rise will be proportional to the spatial sampling resolution. Recent evidence shows linearly increasing foveal information^77^ and also simultaneous parallel information accumulation at the peripheral target without loss of information or interaction with task difficulty.^78^^,^^79^^,^^80^

When the target steps to eccentricity, *e,* (Figure 2C, top), the rate of visual information being gathered about the target feature falls from *r*_*0*_ to *r*_*e*_, and returns to *r*_*0*_ after the saccade re-foveates the target. No information is considered to accumulate during the saccade duration—a model simplification discussed in the Limitations section, but with some support.^79^ If no saccade had been made (dotted line), information would have accumulated via parafoveal preview over the shaded period, and a fixation strategy would gather more information than a saccade strategy during this period before the “payoff-time” is reached. After this “payoff-time,” the saccade choice has always gained more information. Importantly, the time for a saccade to payoff in information gathering is much longer than the saccade duration when *r*_*e*_ is close to *r*_*0*_. When the target steps to a more eccentric location, the rate of information being gathered about the target falls more steeply, leading to the payoff-time reducing toward the saccade duration.

In this scheme, for given stimuli and locations, the payoff-time is known (or in principle knowable), but the information gain needed (*dI*) before another new stimulus may require responding to is typically unknowable, in principle and practice. However, a long payoff-time encompasses more potential bounds in favor of maintaining fixation than moving (Figure 2D), and vice versa for short payoff-times (Figure 2E). The probability ratio of an information gain, d*I*, being achieved by fixation rather than by moving will be monotonically proportional to payoff-time. *P(I|H*_*fixation*_*)/P(I| H*_*saccade*_*)* ***α*** *Payoff-time*.

Thus, the payoff-time can form a convenient “equivalent decision variable”^81^ for an optimal sequential probability ratio test.^82^^,^^83^ The conventional probability ratio for perceptual decisions^84^ and previous saccade models^6^ is *P(e*_*i*_*|H*_*1*_*)/P(e*_*i*_*|H*_*2*_*),* where *e*_*i*_ is the momentary sensory evidence and the hypotheses H_1_ and H_2_ would be, for example, that a target is present on the left versus right. Given fixed sensory stimuli in a trial, but some perceptual input noise, the evidence accumulates on each sample in a diffusion process with the average rate proportional to *P(e*_*i*_*|H*_*1*_*)/P(e*_*i*_*|H*_*2*_*)*. In our scheme, the decision is not between two possible target locations, but between two behavioral responses: fixation and saccade, with the accumulation rate to the saccade decision being inversely proportional to payoff-time.

In sum, if we can estimate how payoff-time varies with eccentricity, we can use this to modulate the rate of the Figure 2A response linear accumulator, and predict how SRT varies with eccentricity. From the geometry of Figure 2C, we have *Payoff-time = Saccade Duration/(1-r*_*e*_*/r*_*0*_*).* Note, here, the numerator is the movement cost and the denominator is a normalized resolution benefit of movement. Payoff time represents a cost/benefit ratio.

### Central (foveola) peak experiment

We computed payoff-times using visual resolution and saccade duration functions of eccentricity under the framework of Figure 2C. The visual resolution in the central fovea matches the cone spacing limit,^75^ and we used best estimates of this from the literature,^85^ while measuring saccade duration and reaction time in subjects responding to the movement of a single pixel target (0.04°) stepping by 0.1–9° under monocular conditions with head stabilized via a bite-bar (see STAR Methods). By recording the right eye, rightward movements were abducting saccades, and leftward adducting. To allow for potential kinematic differences^86^ (or differences in nasal versus temporal retina, respectively), we modeled each direction separately below.

**Figure 3:**
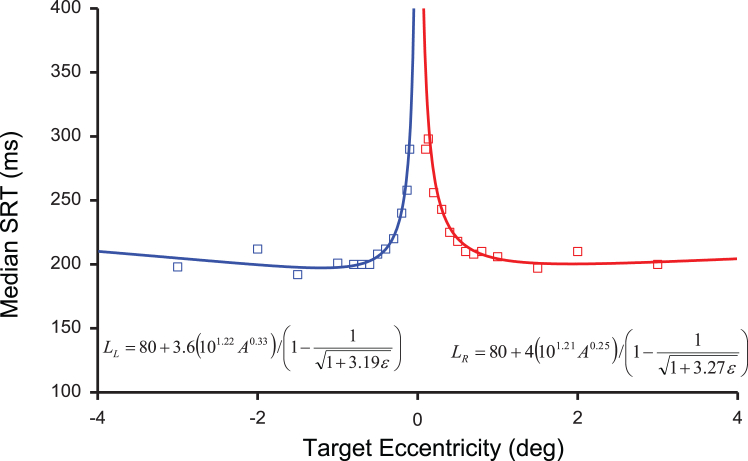
Payoff model fitting to data from point target stepping within the fovea (Experiment 2) Individual median SRTs were averaged across participants at each eccentricity. Because the left eyes were patched, leftward movements were nasal (blue squares) and rightward were temporal (red squares). The SRT (Latency, *L*) equations from the model show the eccentricity, *e*, and the saccade amplitude, *A*, fit with one free parameter, the scaling constants of 3.6 (blue curve) and 4 (red curve). The equation parentheses contain the saccade duration “costs”, and normalized resolution benefits of foveation as functions of *A* and *e*, respectively. Please see results text and STAR Methods for details.

Subjects had similar SRT-eccentricity functions, and we fitted the one free parameter in our model (proportionality constant scaling the mean decision rate that shifts curves vertically) to the median SRT averaged across subjects in the foveola region (Figure 3). Each direction was fitted with separate proportionality constants and yielded similarly strong significant fits (adjusted-R^2^ of 0.96 and 0.95 for left and right, respectively, *p* < 0.001). Outside the foveola, visual resolution matches midget ganglion cell spacings^75^ and resolution falls off more slowly with eccentricity,^87^ while the cost of saccade duration increases monotonically (Figure 2C). Hence, after hitting a minimum around the foveal edge, payoff-times begin to increase again perifoveally because saccade duration increases while the benefit of parafoveal preview information accumulation, *r*_*e*_ (Figure 2C), approaches a floor limit. Hence, our model not only predicts the central peak, but also predicts SRT will rise with eccentricity beyond the macula in a “bowl-shaped” function of SRT with eccentricity^53^ (Figure S6). Of course, multiple factors will likely be involved. If the target becomes harder to detect in the periphery, delays in identifying its location would also increase SRT, which is the standard explanation for increases at eccentricities beyond the macula.^5^ Larger targets reduce that sensory detection problem, but here we argue that their increased visibility also reduces the need for urgent inspection.

### Payoff-times at different spatial scales

**Figure 4:**
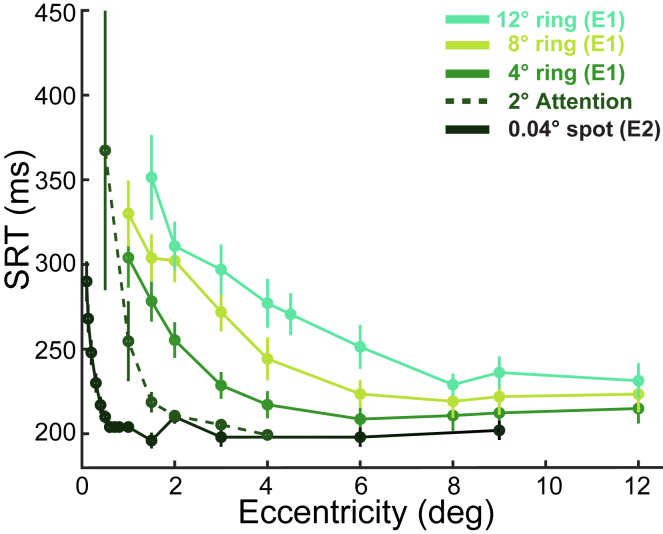
Comparison of SRT curves across target sizes (Experiment 1 and 2) The Experiment 1 data from Figure 1B are replotted, again as mean ± SEM error bars. The Experiment 2 data (Figure 3) were averaged across directions and replotted (black curve for the 0.04° target). To focus on the mean behavior, means and 95% confidence intervals are shown for pooled data, ignoring participant identity in Experiment 2. Averaging across individuals changed the means by < 1 ms on average (range 0–3.3 ms) and increased the confidence intervals by 3 ms on average for eccentricities ≤1°, but increased confidence intervals by 19 ms on average for the infrequent eccentricity conditions >1°. To fill the gap between the point and 4° rings, the 2° ring condition from the previous attention task data (Harwood et al., 2008) is included (dark green). Because we showed that the attention discrimination task in that study invoked considerable “perceptual urgency,” the 2008 plateau values were significantly lower than for our current participants. For easier comparison of the gradual trend in declining SRT-eccentricity slope with increasing target diameter, the attention dataset has had a constant 60 ms added to each eccentricity condition.

An important question, therefore, is whether our model could unify spot-based SRT literature and our spatial scale phenomenon under one payoff-time principle? But first, empirically, we see a family of SRT functions with eccentricity as target diameter increases from 0.04° point targets (Experiment 2) to rings of 2°, 4°, 8°, and 12° diameters (Figure 4). The curves share similar magnitude falls in SRT before hitting a plateau. The steepness of the SRT-decline with eccentricity progressively reduces as target diameter increases, falling over gradually larger ranges of eccentricities.

**Figure 5:**
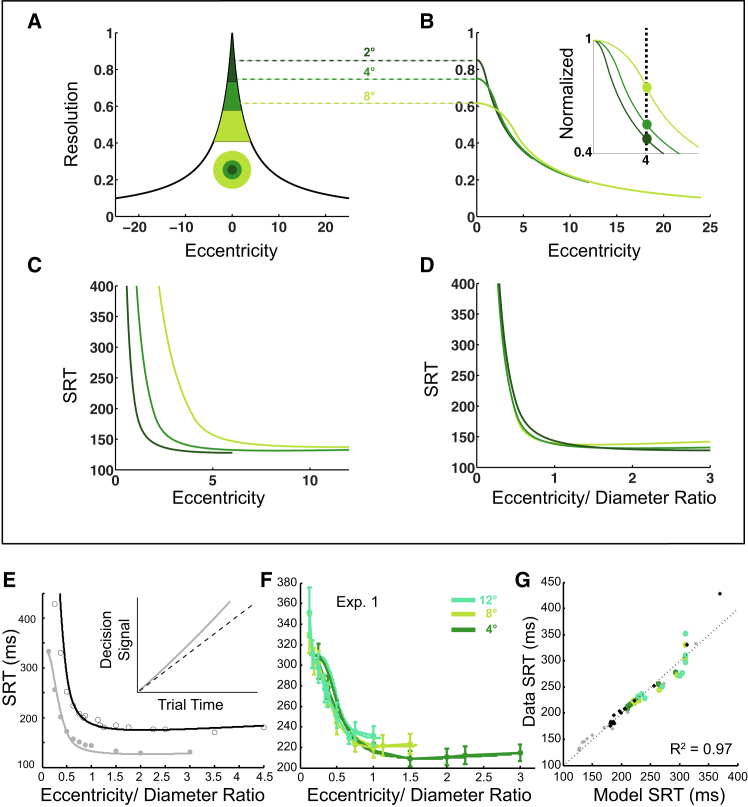
Extending the payoff model to predict SRT from spatially extended targets (A–D) We apply the model framework to extended targets (see results text), showing 2, 4, 8° rings. Panels A-B show resolution curves, and SRT are shown in C and D. (E) Two datasets from Harwood et al. (2008) were fit via either the 1-free parameter model (black curve) or after adding a second free “deadline” parameter of increasing urgency during a trial (gray); the increasing urgency is shown with the rate increasing in the inset. (F) Experiment 1 data (4, 8, 12° rings in mint, olive, and forest green) are replotted from Figure 1B (group mean ± SEM) and fitted to the deadline two-parameter model. (G) All conditions from E and F combined, plotting data and model predictions. Please see results text and STAR Methods for details.

The payoff-time model can replicate these patterns across target sizes. The average resolution of larger targets is lower than smaller targets (Figures 5A and 5B). When an extended target steps eccentrically, its average resolution also declines less steeply (Figure 5B). Because the benefit of refoveation is the increase in resolution once on the central fovea, the relative benefit of different sized targets is computed by normalizing the curves in panel B to the peak resolution for each size target (Figure 5B inset). These refoveation benefits decrease with increasing target size at any given eccentricity, while the costs remain constant because (unlike arm movements) saccade duration is largely unaffected by target size. Thus, larger targets have worse cost/benefit ratios and longer payoff-times, and longer SRT for a given target step amplitude (Figure 5C). When expressing the SRT in terms of eccentricity/diameter ratio, the model SRT curves for each target size collapse onto approximately a single function (Figure 5D). Qualitatively, the same model applied above within the fovea predicts the two key elements of the spatial scale SRT phenomenon: collapsing onto a single curve as a function of eccentricity/diameter, and the sharp increase in SRT at ratios <1.

Quantitatively, our model fitted the data from our previous attention-task paper well^32^ (Figure 5E). Two task instructions were used in that experiment, one with an explicit instruction to saccade on each trial (gray, closed circles) and one where completing the perceptual task was paramount (black, open circles). The model of Figures 5A–5D fits the latter well (black curve); fitting the payoff-time proportionality constant accounted for 97% of the variance in the medians. The explicit movement-instruction (gray circles) was fit slightly less well by this 1-parameter model (not shown, adjusted-*r*^2^ = 0.95). However, because these subjects were instructed to make a saccade on each trial, this task instruction may have acted as a deadline process. An enforced deadline has previously been modeled as a collapsing bound on the decision process,^88^ or equivalently as an increasing rate during the trial (Figure 5E, inset). Adding a fixed increase in decision rate as a function of trial time (i.e., a second free parameter), improved the fit (adjusted-*r*^2^ = 0.99; gray curve in Figure 5E).

Fitting the original payoff-time model with a deadline factor also explained a high proportion of variance from Experiment 1 (Figure 5F) (adjusted-*r*^*2*^ = 0.90). Indeed, this model also captured the 12° eccentricity departures from overlapping curves that were discussed above for Figure 1C (equivalent to the eccentricity payoff-time increase for spot targets beyond 10° mentioned above, Figure S6). Comparing all conditions from Experiments 1 and 2, and the previous attention-task study, showed good overall fits between the model and SRT (Figure 5G, *r*^*2*^ = 0.97).

In summary, the model we propose has the saccade system waiting longer in motor preparation (lower response priorities) when the information-gain benefit of foveating an eccentric target takes longer to payoff. The system exploits the current fixation with information gathering in its periphery and trades this off against the increased information gathering that foveally exploring the target post-saccade would bring. The principle is information gain from the behavioral choice, not evidence accumulation for the choice. This principle explains saccadic post-selection delays more plausibly than sensory-weighted accumulation of evidence, while being entirely consistent with the impressive array of data based on accumulator models.^8^ The architecture of Figure 2C is also consistent with evidence of parallel accumulation of information at the fixation point and at the peripheral target location, with no interaction with task difficulty.^79^ A more stringent test of our approach would also predict volitional choices between two simultaneously present targets (Expt. 3).

### Free choice experiment

**Figure 6:**
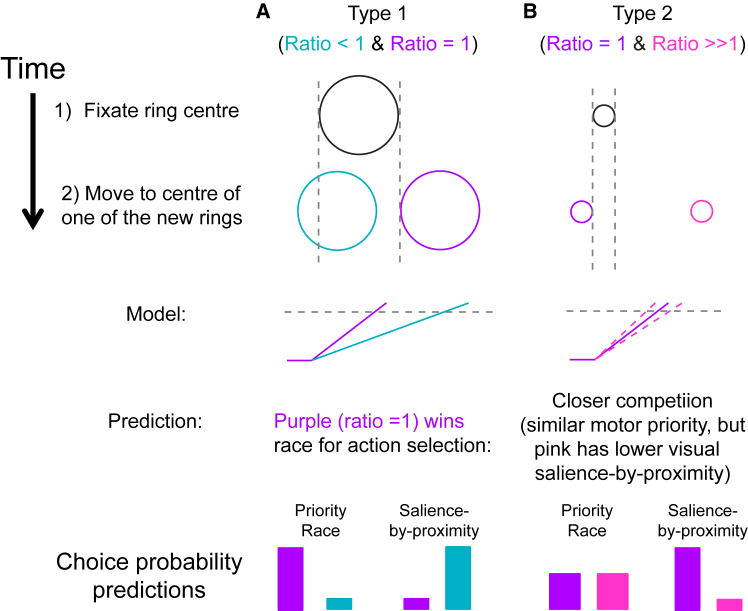
Schematic of experimental design and predictions for Experiment 3 Top panel: participants were instructed to fixate the center of a ring (2, 4, or 8° diameter); following a random interval (800–1300 ms), the fixation ring disappeared, and two rings of the same diameter appeared simultaneously displaced to the left and right of the screen center. One ring was always centered on an eccentricity equal to that of the ring diameter (Ratio = 1, purple). (A) The second ring was at a smaller ratio (0.125 or 0.25; “Type 1 trials”). (B) The second ring was at a larger ratio (4.5 or 9; “Type 2 trials”). Bottom panel: decision signal accumulations as predicted from Experiment 1. In Type 1 trials, the ratio = 1 target wins an independent race to threshold and, thus, predicts that this ratio target is the chosen saccade target; however, variability in the decision process may allow a small fraction of blue trials to “win the race for selection.” In Type 2 trials, the decision rates are much more similar, predicting a more equal probability of choosing the ratio = 1 target, but these nearer targets may still be preferentially chosen due to their greater visual salience-by-proximity compared to the more distal (pink) target. Note that all rings in the experiment were, in fact, red.

We gave 12 naive participants a free choice between two ring targets at different eccentricities. In each trial, one central ring was split into two identical rings to the right and left of fixation. The rings never overlapped (Figure 6). One ring was always at an eccentricity/diameter ratio of 1; the other “competing ring” appeared either nearer (“Type 1” trial) or farther (“Type 2” trial). Instructions were to pick one ring to move to while attending the whole ring, emphasizing that there was no “right” or “wrong” choice and discouraging strategising. To model the new participants’ data, we also ran a preliminary single-target session with the same eccentricity and diameter combinations as in the main choice experiment.

Motor priority and visual salience-by-proximity conceptions of saccade choices give very different predictions in this design. Visual salience-by-proximity arguments predict the nearer target to be chosen most often. Indeed, Findlay^89^ (1980) used saccade preferences to nearer targets to quantify salience. However, our priority of motor response arguments predict preference toward the minimum payoff-time target. Each target had its own saccade-fixation payoff-time prioritization represented as an independent accumulator. Different targets have response accumulators racing to a threshold. In the schematic Figure 6A (middle), our priority model strongly predicts choosing the farther target; variability in each competing accumulation, and free will or alternative cognitive biases, would reduce the choice preference below 100% to the far target. In Figure 6B, the payoff times are similar for both targets, predicting close-to-chance choices. However, salience-by-proximity predictions are again very different, predicting a strong preference for the nearer target.

**Figure 7:**
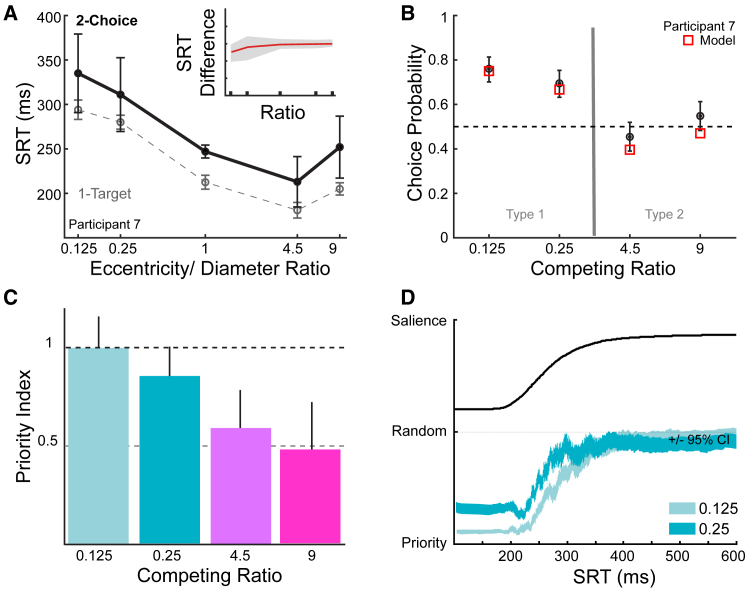
Free choices between 2 horizontal targets are driven by saccadic priority (A) 2-choice median SRTs were ∼40 ms higher than 1-target conditions at each eccentricity/diameter ratio in this typical subject (inset: group average difference with shaded 95% confidence intervals is flat across ratios). Figure S7 expands on the distribution and mechanism for the 2 versus 1 target conditions. (B) Probability of choosing the target at ratio of 1 for each competing ratio condition (black mean with binomial 95% confidence intervals for this example Participant 7; red square is Priority model prediction). Importantly, the “Salience-by-proximity” model would predict 0 for the two conditions left of the gray partition line, and 1 for the two conditions to the right of the divider. (C) Priority index indicates the proportion of trials based purely on the Payoff Priority model (see STAR Methods and Figures S8 and S9 for more explanation of the index) for all subjects at each condition plotted as means ±95% confidence intervals. (D) Choice probability as a function of SRT in two ratio conditions (light blue: 0.125 competing ratio; dark blue: 0.25). The colored 95% confidence bands show that participants were more likely to choose the “Priority target” over the “Salience-by-proximity target” at shorter SRT. The corresponding individual participant data are shown in Figure S10. Solid black curve shows the cumulative distribution of SRTs across all subjects with an arbitrary vertical offset (to show the low proportion of trials with SRT <200 ms or >350 ms). Longer SRTs have choice preferences closer to chance and probably indicate greater involvement of top-down cognitive factors.

Median SRTs showed the same pattern as in Experiment 1, collapsing on a function of eccentricity/diameter (Figure S3), but were about 40 ms longer when subjects chose between two competing ring targets, compared with the same movement to a single target (Figure 7A). The increase was approximately constant across the five ratio conditions, as shown by a typical subject (Figure 7A) or by taking the difference between 2-choice and 1-choice SRTs at each ratio for each subject (Figure 7A, inset). Although there was some intersubject variability at the lowest ratios, the average SRT differences were flat across conditions at an average of 37 ms (bold curve, Figure 7A, inset). This 2-choice increase was expected from Hick’s Law,^90^ in which increasing alternative choices increases response times generally. Typically, Hick’s law is understood as each choice accumulating from a lower likelihood starting level, thus taking longer to reach a response threshold. We tested that mechanism for the SRT increase, as well as a pure delay and mutual inhibition between accumulation rates^91^ (see STAR Methods). We found unambiguous evidence for the mutual inhibition of accumulation rates (Figure S7) as opposed to a reduced starting level.

Choice probabilities matched payoff-time prioritization, not salience-by-proximity, predictions. Probabilities of choosing the ratio = 1 target (“CP1”) as opposed to the competing target are shown for a representative subject (Figure 7B), together with this subject’s model prediction (red squares). The predictions used the SRT rate and rate variability inferred from the single rings condition (plus individual mutual inhibition factor). Thus, despite the free choice and potential cognitive biases, this participant approached a 100% “Priority preference” given that departures from the highest priority target were accurately predicted from their SRT variability (the opposite choice sometimes winning the race for selection against the priority odds). For example, the mean choice preference in the 0.125 condition of 76% to the highest priority distal target closely matched the priority model prediction of 75%. Remarkably, the free choice has not lowered their mean preference below the pure-priority model toward chance.

Importantly, the data are far from the 100% salience-by-proximity prediction in all the conditions. Salience-by-proximity choices which would predict CP1 = 0 (not CP1 = 0.5) in the 0.125 and 0.25 competing target conditions, and CP1 = 1 in the 4.5 and 9 competing ratio conditions. The data and models were collapsed across the 3 eccentricities at each ratio, therefore these choice biases do not represent simple amplitude biases. Some participants were more likely to choose near or far targets, but there was no proximity bias overall (Figure S8A). Because all targets were along the horizontal meridian, we did not expect the known anisotropies in saccade reaction^92^ and prefences^93^^,^^94^ to influence our data. However, we did expect some participants to have directional biases due to effort-saving default strategies or due to the reported rightward biases in some studies.^95^^,^^96^ Five participants were significantly biased, preferentially choosing rightward targets, leading to an overall group rightward bias. This bias was independent of ratio, allowing us to correct for this in a less biased “Priority Index” (see Supplemental Information, Figures S8 and S9). A Priority Index of 1 indicates a subject choosing the higher payoff target every trial, once the variability in accumulator races (and any directional bias) is accounted for. Priority Indices >1 mean that a subject chooses the higher payoff target more often than two racing accumulator models predict (e.g., despite a bias to the right, often choosing a leftward high-payoff target). Salience-by-proximity predictions have a Priority Index equaling zero. Remarkably, the average Priority Indices in the 4 conditions were 1.00, 0.85, 0.58, 0.48 (Figure 7C); the first two were not significantly different from an index of 1 (*p* > 0.05), and all conditions were highly significantly biased away from the salience prediction (*p* < 0.001). Payoff prioritization dominates saliency-by-proximity in our design.

Finally, salience-by-proximity had an even weaker impact on faster SRT. Salience is typically thought to act faster than processes related to the behavioral goal.^97^ In contrast, even subjects with lower priority indices, were more likely to choose the high payoff-prioritization choice at short SRT (Figure S10), with their choices becoming more random at longer SRT. The group level for the two key conditions (Figure 7D) was significantly below chance choice behavior, toward payoff-prioritization, until >350 ms.

## Discussion

These findings firmly establish simple object size as a key determinant of human saccade latencies and offer an explanation to the enduring mystery of saccadic procrastination. Simple targets stepping by less than their diameter (eccentricity/diameter ratio <1) have systematically delayed saccade reaction times. This one parameter explained up to 97% of median SRT variance at ratios up to 1 (Experiment 1). The new payoff-time model of saccade decisions linked this effect of object-size to the long-known “central peak” in SRT within the fovea (Experiment 2), providing further support that microsaccades form a continuum with larger saccades. Crucially, the model finally explains why procrastination occurs after target selection. The wider relevance of the remarkable structure and strength of this phenomenon can be seen in its persistence in a cognitive “free choice” task (Experiment 3), in its potential applications, and its neural correlates in monkeys (see companion article^60^).

Given decades of research into the saccade system, it is remarkable that this central feature of saccade decisions has been so overlooked. Most saccade studies have focused on the plateau region of the behavior (ratios >1). Typical eye movement designs feature small spots to control position precisely, for which ratios <1 equate to eccentricities within the foveola. Within the foveola, there is a clear “central peak” in SRT that has been shown to be of motor preparation origin.^52^^,^^53^^,^^54^^,^^55^ Other studies have only dipped into the non-plateau region^46^^,^^47^^,^^48^^,^^49^^,^^54^ and have, thus, reported inconsistent findings. Only one previous study has systematically characterised the ratios <1 region, and that was in a specific highly demanding dual attention task, which had correspondingly uncertain ecological or external validity.^32^ Ecologically, saccades are made between simple objects of different sizes. The demonstration in the current study that simple object size determines both SRT and choice behavior greatly increases the likelihood of ecological validity. The increased external validity is shown by the replication of the behavior in naive monkeys (untrained on extended-ring targets, Figure S4).

### A “motor intensity” rule

We interpret the power law decrease in SRT with increasing eccentricity/diameter ratio (Figure 1D) as a “Motor Intensity” rule, by analogy with Pieron’s power law between increasing Stimulus Intensity and decreasing response times. The further a target steps into the periphery, the more visual benefit is derived from foveation; more proximal targets get less benefit from foveation and are set a lower urgency for response (lower motor intensity, lower “motor priority”). Importantly, the obligatory nature of this motor intensity rule is seen by attempts to invert it via explicit reinforcement (rewarding faster SRT to large targets and slower SRT to small targets).^68^ We found that SRT was only modestly susceptible to these explicit cost/benefit modulations over thousands of trials. In the payoff-time model framework, this is consistent with an ingrained learning of the cost/benefit statistics of the oculomotor system, and the largely fixed main sequence costs and resolution benefits of saccades.

The parametrization of this Proximal Procrastination (or, conversely, distal “motor prioritization”) allows for graded experimental control of SRT. Classic manipulations of motor preparation have relied upon discrete manipulations. For example, varying target location probabilities across blocks,^6^ or the number of possible targets present.^44^ Fixation-offset timing relative to target onset (−200 ms “Gap”; 0 ms; no offset “Overlap”) has also been argued as effecting heightened-to-reduced motor preparation from Gap-to-Overlap.^98^ These fixation-offset conditions can be seen as additive on top of Proximal Procrastination (Figure S2B) (see also^55^ for equivalent additivity in the “central peak”). Top-down Priority tasks are also typically discrete manipulations.^38^

### Experimental utility of parametric control of saccade reaction time preparation

Many potential experiments become possible with parametric control over saccade preparation. For example, countermanding saccades paradigms rely on inferring “stop-signal RT” from distributions over trial blocks.^99^ Controlling the Go-signal response (and perhaps the Stop-signal) on a trial-by-trial basis would make the causal link more direct, especially if neural correlates exist on a similar refined scale. Instead of using Delayed Saccades paradigms to study visual and motor processing separately, proximal procrastination should facilitate studying visuomotor integration via gradated segregation. Given the existing detailed knowledge gained from discrete manipulations of saccade timing,^14^^,^^98^^,^^100^^,^^101^^,^^102^^,^^103^ finer parametric control should allow for further physiological and modeling advances.

The first physiological study to systematically explore the proximal procrastination region with spatially extended targets^60^ gives direct evidence that eccentricity/diameter scales the saccade preparation “build-up neurons” of the superior colliculus, among other novel decision mechanism findings. This companion article reveals remarkably strong trial-by-trial neural correlates in individual superior colliculus neurons. Given some redundancy across saccade circuitry, other subcortical or cortical areas are likely also modulated by eccentricity/diameter ratio.

Proximal Procrastination’s effect size is another key empirical strength. SRT differences in excess of 100 ms, and non-overlapping distributions (Figures 1D and 1E), are useful for future experimental designs, and make this the largest reported systematic effect for simple suprathreshold-contrast objects in the SRT literature. Of course, lowering contrast toward threshold also has strong effects on the target detection part of SRT.^41^^,^^64^^,^^65^ Note that the large median differences and relatively low variability in Experiment 1 may be partly due to using “non-ageing” foreperiods. Most saccade experiments introduce unnecessary variability from timing-expectancy via uniform random fixation periods prior to target onset.^104^ However, Proximal Procrastination is not reliant on foreperiod timing, as uniform foreperiods were used in the other experiments.

Parametric control of gaze reaction time might have application outside the laboratory. For example, speed/accuracy trade-offs of movement duration (Fitts’ Law^105^) have been widely applied in human-computer interaction.^106^ Manipulations of reaction time via the eccentricity/diameter of stimuli might also be useful.^107^ If gaze choice behavior is manipulable as suggested by Experiment 3, there might be other applications. The magnitude and consistency of the behavior could also lend itself to useful clinical application, given the frequent importance of saccades to neurological investigations.^108^^,^^109^^,^^110^^,^^111^

### Response mode selection: Informational-gain likelihood ratios

Optimal sensory-based decision models operate via accumulating sensory weight-of-evidence (log likelihood ratios) for two opposing hypotheses (e.g., motion coherence, or target location, left versus right).^6^^,^^81^ Constant stimuli lead to linear mean decision rates (with or without drift diffusion). Our key explanatory change is recasting the SRT decision variable likelihood ratios as the likelihood of achieving any given information gain via fixation (peripheral-target preview) versus via saccade (foveation). All SRTs then become “choice reaction times” between the dichotomous response modes, fixation-movement. Doing nothing is a choice that still accumulates peripheral-target information. We argue the key motor decision is not response selection, but response-mode selection. A similar conceptual change has also been made in a search model.^112^

The conceptual change explains the enduring mystery of reactive saccade procrastination.^9^^,^^113^ A single high-contrast target on a blank background has no real visual selection likelihood ratio, nor need for motor selection—there is only one target; thus, task-instructed participants should proceed with minimal procrastination or variability. Conversely, the likelihood ratio of gaining further target information from fixation or saccade is always present and will always depend on the eccentricity of the target. If the amount of information required were known, the optimal fixation/saccade response mode would again not require a decision, and the procrastination enigma would remain. But “required information” will typically be unknown, blurring the best decision and adding delay variability. Adding saccade opportunity costs of reducing information about the opposite side of space, which may be more ecologically vital (e.g., a leaping leopard), should bias the optimal decision for single targets toward a fixation-choice response mode, adding procrastination.

### Model comparisons

An important model strength is that ours builds on, rather than replaces, past models. The many Carpenterian successes, including importantly describing reading and search saccadic behavior,^8^ stand but with arguably a firmer foundational likelihood ratio. Similarly, our model is consistent with fixate-move mechanistic models of SRT^103^^,^^114^ and their physiological balancing generation.^56^^,^^115^ Search and reading models naturally include fixate/move switches and peripheral-preview information considerations that describe their data well.^112^^,^^116^^,^^117^^,^^118^^,^^119^^,^^120^ Optimization search models based on information gain also exist.^121^^,^^122^^,^^123^ But we do need a new model for reactive saccade procrastination, because of: (1) the conceptual problems with the decision signal discussed above; (2) the new Proximal Procrastination data; and, (3) reactive saccades are an ideal controlled paradigm to study neurophysiological correlates, which then inform more cognitive tasks.

Unlike previous reactive-SRT models, the information processing benefit of peripheral preview is inherent in our payoff-time model, but this has the knock-on effect of increasing SRT for eccentricity/diameter ratios <1 (“proximal procrastination”). In sequences of saccades, if peripheral preview also visually selects the “n+1” saccade, some or all of its target selection time (fixed at 80 ms in our isolated targets design) may be eliminated. Proximal Procrastination effects may be more visible in the first saccade in a sequence, with Proximal Procrastination masked to an extent in future intersaccadic intervals by savings in target selection time.

### Incorporating cognition and predicting free choices in ratio races

Is there a place for proximal procrastination in naturalistic behavior such as visual searching? As a first, maximally controlled, approach to volitional cognition, we simply gave a free choice between two targets. Sensory-weighting conditions were pitted against motor-waiting (“priority”) conditions. Classic choice reaction times between multiple targets require separate decision units for each target, which compete in a race-to-threshold with winner-takes-all.^8^^,^^124^ Considering that decision variability inevitably results in “weaker” stimuli sometimes winning likelihood-ratio races, choices matched priority predictions remarkably closely (Figure 7).

The Proximal Procrastination payoff-time model promotes eccentricity/diameter ratios around 1, because the payoff-time tends toward a minimum at those ratios, and larger ratios require longer movements. Thus, a good strategy during visual search might be to make saccades of about the same size as, or just larger than, the spatial scale at which one is attending. Evidence consistent with this has been found in the monkey, in which saccade size is related to stimulus density in a conjunction search task.^125^^,^^126^ The spatial scale of attention has been shown to affect visual search in humans.^127^ Some search models suggest that saccade endpoints are planned to counter the fall-off in visibility with eccentricity, by choosing new fixations that maximize information accrual.^121^^,^^122^^,^^123^ From our cost/benefit logic, we would expect that these “most informative landing positions” would also lead to the fastest decision times. Turning the argument around, target selection in a complex environment may involve maximally informative areas having decision rates that are faster to reach threshold, which results in their becoming saccade endpoints; less informative locations having lower rates will lose the competitive race for target action-selection.

### Cost-function optimization versus payoff-time heuristic

While the payoff-time model proposed here is optimal in the same way as Carpenter’s SRT model (an optimal sequential probability test), one might ask why not follow an explicit cost-function optimization as in saccade kinematic models,^128^^,^^129^ or in the search models^121^^,^^122^^,^^123^ above? Given that quadratic cost-minimization is itself an assumption,^130^ only rarely experimentally tested,^131^ optimization models that rely purely on data,^132^^,^^133^ potentially, survive Occam’s razor better. Our basic model attempts to stick closely to data and has only one free parameter (a constant of proportionality). Moreover, the model is biologically heuristic in terms of allowing the saccade system an easy shortcut to make decisions based on the value of time and information gathering based simply on the size and distance of a target.

Ultimately, the strength of the empirical data presented here and in the neurophysiological recording data in the companion article^60^ demand an explanatory theoretical model. If these data help establish a fundamental property of “active vision”, multiple, potentially better, explanatory models may be forthcoming. As an initial step, the payoff-time model offers the first explanation for how saccade preparation rates vary across the visual field, providing a crucial link between the known “central peak” of foveal latencies and object-targeted SRTs. Arguably, the payoff-time model gives the most plausible explanation yet for the enduring enigma of saccade procrastination and why single targets on blank backgrounds have such long and variable SRT.

Intriguingly, Roger Carpenter mused that the underlying explanation for the procrastination and variability could be deliberate injection of randomness in a game-theoretic disruption to encourage visual exploration.^134^ In deference to his seminal work on saccadic decision making, we muse that payoff-time represents an equilibrium point between strategies of fixation and saccade. Game theory requires that the value of different outcomes are measured on the same scale.^135^ Payoff-time translates the two key functions of saccades, resolution improvement and speed, into a common time-based decision metric. Evidence for Nash equilibria exists within the saccade system.^136^ Could populations of neurons across the hemispheres be competing against each other with their two strategic choices of fixation (exploitation) and saccade (exploration)? Proximal Procrastination discourages exploring areas that have poorer cost/benefit ratios.

### Exploring sensorimotor preparation

The elephant in the room of sensorimotor decision-making has been that we have too little understanding of why motor preparation is so long and variable. As scientists, we naturally ascribe causality to things we can manipulate, focusing on the sensori-part and lumping much else into “non-decision time.”^137^ Our study demonstrates that object relative eccentricity is a trivial trial-by-trial tool to manipulate motor preparation, which should greatly facilitate further exploration of motor preparation. Ironically, the best way to explore this elephant may be with the eccentricity-to-diameter ratio that discourages saccades to targets that are near and very easy to see peripherally.

### Limitations of the study

A diffusion model with non-decision time that changes as a function of eccentricity/diameter would descriptively fit the data at least as well as the payoff-time model does here. However, the diffusion model would still need an explanation for how the non-decision time changes with eccentricity/diameter ratio. The advantage of the payoff-time model is that it prescribes the mean decision rate itself as a function of eccentricity/diameter. (Without a non-decision time factor, we have shown that diffusion or linear accumulator models fit our monkey SRT data equally well.^60^).

Similarly, a simple mechanistic explanation based on lateral inhibition in the superior colliculus might explain the behavior, but that would still leave the question as to why the inhibition is wired in this specific way? A mechanistic explanation is crucial, but can be seen as a complementary implementation-level explanation without necessarily excluding a computational level explanation such as payoff-time.^138^ Note that our current physiological data are from single cells,^60^ rather than population “point images,”^139^ and previous neural network models of colliculus generation of SRT were essentially focused on the standard, plateau region of SRT.^102^^,^^103^ Thus, it remains to be seen if collicular lateral inhibition can replicate, or explain, the proximal procrastination data.

The payoff-time model makes various assumptions that can be challenged. First, it assumes that no precision information is accumulated during a saccade (Figure 2C). While saccadic suppression degrades the visual information during a saccade,^70^^,^^140^ and there is data consistent with no task-related information during a saccade,^79^^,^^141^ it is also incomplete.^142^ Importantly, the payoff-time model is intended as a “First-order model.” Future data can refine (or refute) it. If the slope in Figure 2C fell to non-zero information accumulation during saccades, it would shorten payoff-times for both proximal and distal targets, but payoff-time would still be longer for proximal targets; the organizing principle for procrastination could remain.

Second, at the weakest motor intensities (lowest eccentricity/diameter ratios), it was assumed participants implement a deadline process due to the instruction to make a saccade on each trial, which added a second model parameter to reduce SRT. Without this movement instruction, the payoff-time model may have fit elevated SRT better in the original one-parameter model (Figure 5E). The volitional urgency was modeled with an accelerating rate signal during the trial, rather than the more typically used collapsing bounds.^143^ Although rate urgency and collapsing bounds are often seen as equivalent,^144^ the payoff-time framework and the variable bounds found in our monkey data suggest that a collapsing bound mechanism may have been more appropriate.

Third, a fixed visual selection time was used in the model, which was independent of target size (see stage-1 diffusion process in Figure 2A). Based on past literature, this was set at 80 ms.^39^^,^^40^^,^^66^ How plausible is this? Increasing this visual selection window to 100 ms would simply require a small decrease in the proportionality constant of the subsequent (Proximal Procrastination) stage in the model, shifting the SRT-eccentricity curve to offset a longer visual selection period. Whether future studies will reveal a small size-dependency of the visual selection window, necessitating an additional parameter in the model, is an open question. The likely direction of change would be to increase the importance of Proximal Procrastination, because larger targets might expedite visual selection processing. Currently, the evidence for expedited visual transients for larger targets has only been reported in the colliculus at lower contrasts than used here.^67^

Finally, the Free Choice experiment was geared to the novel proximal (Type 1) trial conditions and should be tested with a broader range of conditions in future studies. Related past studies have all used Type 2 conditions with competing choices all at eccentricity/diameter ratios >1. As above, the payoff-time model prefers ratios ∼1, since larger eccentricities carry longer duration costs, corrective movement costs,^73^ as well as signal-dependent^128^ or other forms of “effort” costs.^145^ These higher-order costs would tend to bias choices in Type 2 trials to nearer targets, which may explain the proximity-preference in recent Type 2 studies,^146^^,^^147^ and from the older Findlay^89^ (1980) study. It would be interesting to incorporate potential direct measures of these higher-order costs by analysing pupil diameter during saccade preparation.^148^ Pupil diameter has been linked to the effort of planning saccades,^94^^,^^145^^,^^149^ and would be expected to change alongside eccentricity/diameter ratios in future studies.

## Resource availability

### Lead contact

Requests for further information and resources should be directed to and will be fulfilled by the lead contact, Mark Harwood (m.harwood@uel.ac.uk).

### Materials availability

This study did not generate new unique reagents.

### Data and code availability

• Data: Eye movement data have been deposited at Mendeley Data and are publicly available as of the date of publication at https://doi.org/10.17632/vhrtsntnvd.1
• All original code is available from the lead contact upon request.
• Any additional information required to reanalyze the data reported in this article is available from the lead contact upon request.

## Acknowledgments

This research was supported by a 10.13039/100000001National Science Foundation grant (1232654) to Mark Harwood (PI). Warm gratitude goes to Annabelle Blangero for all her input and to Laurent Madelain for many valuable discussions. Thanks also goes to Jay Edelman for the use of his bite-bar eye tracking setup for Experiment 2, and to the reviewers for their helpful comments to improve the article.

## Author contributions

M.R.H. designed, ran and analyzed all experiments and models, and wrote the article.

## Declaration of interests

The author declares no competing interests.

## STAR★Methods

### Key resources table

**Table:** 

REAGENT or RESOURCE	SOURCE	IDENTIFIER
**Deposited data**		

Eye movement data	This study	https://doi.org/10.17632/vhrtsntnvd.1

**Software and algorithms**		

MATLAB	Mathworks	R2021b
Custom Code	This study	N/A
Psychophysics Toolbox (VideoToolbox) Version 3.0.19	Psychtoolbox	http://psychtoolbox.org/download
*Vision Shell PPC (C custom Software libraries)*	Raynald Comtois	N/A

### Experimental model and study participant details

We recorded 20 unique participants across the experiments presented here (Ages: 19-51 years; 10 Female, 10 Male; 5 non-white; all either had postgraduate qualifications or were studying towards a BSc, MSc, or PhD). Each participant showed the same statistical effect of eccentricity and target size on SRT (see below and, for examples, Figures S1–S3). Because the core SRT phenomenon was present for each individual participant, and because reflexive SRTs are typically not thought to be strongly influenced by individual differences, we did not design the studies with sufficient power to investigate individual differences, and no such analyses were conducted. Participants were unpaid and recruited by opportunity sampling with the only exclusion criterion being known neuro-ophthalmological existing conditions. No participants were excluded post-recruitment (for trial exclusions see data analysis section below).

In Experiment 1, six participants were recorded (ages: 32-51 years, mean = 44.2, SD = 7.0; 3 Female). Two had previous experience of eye movement experiments. The additional data shown in Figure S2 were recorded from another six participants (one overlapping from Experiment 1) (ages: 25-41, mean = 33.1, SD = 6.4; 3 Female); five had prior eye movement experiment experience. In Experiment 2, which required head stabilisation via a bite-bar and 1560 trial recordings (see below), we recorded three participants (ages: 30-35, mean = 32.6, SD = 2.5; 2 Female); all were experienced eye movement participants. In Experiment 3, 12 participants were recorded (ages: 19-35 years, mean = 26.6, SD = 5.1; 6 Female). Eight of these participants were familiar with eye movement experiments, but none were familiar with the specific design of Experiment 3 and there was no overlap in participants between Experiments 1 & 3 (Figures 1 and 6). All procedures were approved by both the City College of New York Institutional Review Board, and the University of East London Research Ethics Committee, and informed consent was obtained from every participant prior to recordings.

### Method details

#### Apparatus

Two laboratories were used during data collection. Experiments 1 & 3 were recorded using an Eyelink-1000 eye tracker (SR Research, Osgoode, Ontario, Canada) with head stabilised via chin and head rests while viewing a 19-inch CRT display at 57 cm; red ring stimuli (25% Weber contrast) were presented on mid-grey backgrounds via Psychophysics Toolbox^150^^,^^151^ in Matlab (The Mathworks, Natick, MA, USA) at 120 Hz frame rate and 800 x 600 pixel resolution. Experiment 2 was recorded using an Eyelink-II with head stabilised by bite-bar while viewing a CRT display at 60 cm with white targets on a black background; stimuli were generated by Vision Shell software (Raynald Comtois) at 85 Hz frame rate.

#### Behavioral tasks

##### Experiment 1: Eccentricity and object size

*Experimental design*: the key dependent variable measure was ‘reflexive’ saccade reaction time (SRT) to steps of single ring targets. Two variables (ring target eccentricity and diameter) were manipulated with the aim of producing a wide range of eccentricity/ diameter ratios. The great majority of naturally occurring saccades are to eccentricities < 15°.^152^ Objects targeted in the real-world are typically much larger (>1°) than targets used in conventional reflexive SRT paradigms (<1°). To investigate more natural ranges of object size and eccentricity than has been commonplace, we used eccentricities between 1-12° and three ring diameters (4, 8, and 12°). Because our interest was in SRT relative to the ratio of eccentricity/ diameter, we selected 27 conditions to produce a finely graded range of 15 eccentricity/ diameter ratios between 0.125 and 3. All three rings were presented at eight eccentricities (1.5, 2, 3, 4, 6, 8, 9, 12°); the remaining 3 conditions were the two smaller rings presented at 1° eccentricity (ratios = 0.25, 0.125) and the largest ring at 4.5° eccentricity (ratio = 0.375) (to allow comparison across all three rings at a ratio in this important region between 0.25 and 0.5 ratios). The 27 conditions were each repeated 24 times, generating 648 trials for each participant. Trials were run in 18 blocks of 36 trials.

*Stimulus design:* Visual stimulation of the foveal region can inhibit SRT (‘Overlap’ paradigm^153^^,^^154^) and peripheral visual transients can also transiently inhibit SRT (‘remote distractor effect’,^155^ ‘saccadic inhibition effect’^156^). To minimize foveal visual stimulation and visual transient impact on SRT, we chose thin ring stimuli for Experiments 1 & 3. Arguably, a thin ring may help attention ‘stick’ to one spatial scale. Control data with solid circles of 4, 8 or 12° (Figure S2C) showed that this design concern has apparently no material effect.

*Trial structure:* Each trial began with a small black fixation spot (0.3°) at the screen center. Participants were required to be stationary on fixation (+/- 0.5°) for at least 250 ms before the fixation spot was replaced by a fixation ring of either 4, 8, or 12°, appearing still at screen center. This ring remained at 0° for a variable foreperiod (0.62-1.51s), during which breaks in fixation would reset the trial to the black fixation spot. After this fixation foreperiod, the central ring stepped left or right by 1-12° and remained visible until 300 ms after a saccade to it, after which the trial ended. Online detection of anticipatory saccades (reactions < 80 ms) ended a trial early, replacing the eccentric ring with a 1° blue spot, which remained on for a “penalty” 1s, before the next trial began with the black fixation spot at 0°. There was only ever one visual stimulus on screen at any time during the trial.

*Spatial randomisation*: All trials started at screen center to be in the naturally predominant primary position, and to have direction randomised without any prediction available from screen edge starting position, and to reduce potential effects on SRT of sequences in one direction.^157^ Directions were pseudo-randomised across each block of 36 trials (18 leftward and 18 rightward).

*Temporal randomisation:* ‘Non-ageing’ foreperiods were chosen to reduce timing predictions adding to SRT variability. Conventionally randomised (uniform distribution) fixation periods of standard reflexive SRT paradigms induce an increasing expectation within the fixation period of the impending target step. Oswal and colleagues^104^ have shown that this adds considerable SRT variability. To reduce this common design artefact, we followed the Oswal approach in having an exponentially weighted foreperiod distribution (more short fixation periods). This distribution maintains a constant expectation (non-ageing) fixation foreperiod. We chose a distribution to average 1s over each block of 36 trials.

*Task instructions*: Participants were simply told to follow what was on screen, maintaining fixation at the center of the ring until it moved randomly left or right, at which point participants should follow the ring as quickly as possible, regardless of how large or small the steps of the ring. There was no instruction to target the center of the peripheral ring, just to follow it while attending to the whole ring.

*Block structure*: Target diameter was held fixed within each block of 36 trials, allowing all 9 eccentricities to be pseudo-randomised within a block, presenting 4 of each eccentricity. This was designed to make the stimulus conditions less jarring for the mostly inexperienced participants. Blocking is not critical to the experiment (Experiment 3 used randomly interleaved target diameter on every trial, and produced the same SRT pattern). The block order was pseudo-randomised for each participant. All 18 blocks were recorded in a single session, with participants encouraged to take as long self-generated breaks between blocks as they needed to maintain their attention. Participants were also told that each trial would only start when their eyes were open on target, so occasional within-block breaks were possible by closing their eyes. No participant chose to implement their right of withdrawal.

*Eye tracker calibration:* The Eyelink 9-point calibration grid was used to calibrate the right eye of each participant. Calibration was paced by the researcher with each point and overall calibration validated. Drift was automatically checked online at the start of each trial (not requiring ‘spacebar intervention’) and between blocks. Calibration was repeated, if necessary.

*Gap, Step, Overlap conditions:* Given that we argue that our SRT effects are at a post-target selection, motor preparation stage of the saccadic decision process, additional recordings were made using the dominant paradigms used to study saccadic motor preparation over the past 50 years: the ‘Gap’ and ‘Overlap’ effects.^153^ Five participants experienced in eye movement experiments were recorded in these conditions with the ‘Step’ condition having the same trial structure as in Experiment 1, but for only six ratio conditions. Pseudo-randomised blocks of ‘Gap’ or ‘Overlap’ conditions had trial structures with either a 200 ms offset of the central ring target before its eccentric reappearance (‘Gap’), or the appearance of the eccentric ring without the offset of the fixation ring (‘Overlap’).

##### Experiment 2: Foveolar SRT peak

*Experimental design:* The aim was to detail how SRT falls with eccentricity across the central rod-free region of the retina that has a 1-to-1 mapping of cones to ganglion cells: the foveola, which extends to approximately 0.7 ° eccentricity. A white square (0.04°) was used as the fixation and saccade target, stepping to eccentricities of 0.1-9°. There were 30 conditions (15 eccentricities x 2 directions). To reduce potential visual detection delays in SRT from the small size of the target, its luminance was bright (78 cd/m^2^) and was presented on a black background (0.013 cd/m^2^). Viewing was monocular with left eyes patched, in order to minimize biological noise from visual eye differences or disconjugate eye movement factors, and to allow a nasal-temporal SRT comparison. Each participant had good visual acuity (6/6); one required recording with their contact lenses to correct for myopia and achieve 6/6 acuity. To minimize head movement noise, heads were restrained via dental impression bite bars. This stabilisation also allowed for a reduction in eye tracker instrument noise by disabling of the standard Eyelink Pupil minus Corneal Reflection recording mode, sampling at 500 Hz in Pupil-Only custom mode.

*Procedure:* Each trial began with the target in the center of the display. After a random interval (800-1300 ms; uniform distribution), the target stepped to the left or right by 0.1- 9°. Each participant was recorded in four sessions on separate days. In the first three sessions, 20 possible eccentricities were presented (+/-0.10 to 0.80° in 0.1 increments, 1.0 and 2.0°) with 20 repetitions to make a total of 400 trials in each session. In the final session, there were 10 possible eccentricities (+/-0.13, 1.5, 3.0, 6.0, 9.0°) presented 60 times at the smallest amplitude (to match the number for eccentricities from the first 3 sessions) and 30 times at the others (360 total trials). Breaks were given every 50 trials in sessions 1-3 and every 60 trials in session 4. Target directions and amplitude were pseudo-randomised across each session. Participants were simply instructed to follow movements of the fixation target as quickly and accurately as possible, no matter how small the target movements. The Eyelink was calibrated via a 13-point calibration grid.

##### Experiment 3: Two-alternative free choice

*Experimental design*: To test if the proximal procrastination has a role in cognitive choice between two stimuli, we followed the same general design as Experiment 1 but with the central fixation ring splitting into two rings of the same size and color placed at different horizontal eccentricities. Three different ring diameters (2, 4, or 8°) were interleaved on different trials. One ring always appeared at an eccentricity of one diameter (2, 4, or 8°), giving a choice between a target at an eccentricity/diameter ratio of 1, and a ring at nearer or farther eccentricities in the opposite direction; hence, there was never any overlap between the rings (Figure 6). Naïve participants were instructed to make a free choice between which target to follow, and that there was no “right” or “wrong” choice or strategy involved. The key dependent variable was choice probability, specifically the choice probability of choosing the ring at ratio = 1 (CP1) over the “competing” choice of ratio (either 0.125, 0.25, 4.5, or 9).

*Procedure:* Before running the Free Choice experiment, each participant completed a session with single rings for the 5 eccentricity/diameter ratios, to enable modelling the decision signal at each ratio for each individual. There were 11 conditions in total covering the 5 ratios (2° ring stepping eccentrically 0.5, 2, 9, 18°; 4° ring stepping 0.5, 1, 4, 18°; 8° ring stepping 1, 2, 8 °). Each of these combinations were repeated 44 times (484 trials total) in a single session. Again, the trial always started in the screen center, and the fixation target ring remained visible for 800-1300 ms (uniform random distribution) before stepping eccentrically. Conditions were pseudo-randomly interleaved across the session. Two sessions of the two-alternative free choice paradigm were recorded. Because there was always a ring at eccentricity/diameter ratio 1 and one other ratio, there were only 8 conditions using the same combinations of rings and eccentricities as in the single-ring session. These were repeated 120 times in each condition, splitting the 960 trials for each participant into two sessions of 480 trials.

*Priority label:* Because of our arguments supporting a motor urgency (‘motor priority’) origin for the effect of the spatial scale of attention^32^ (as well as the empirical manual control data in Wyman and Steinman,^52^ 1973), and the model presented here, we label choices to an eccentricity/diameter ratio of 1 as the ‘Priority model’ choice when competing with lower ratio conditions that have longer SRT (lower priority) (Figure 6A). For trials with ratios >1 (Figure 6B), which have similar SRT (priority) to that at eccentricity/diameter ratio of 1, the Priority Model choice is largely ambivalent, predicting CP! of approximately 0.5.

*Salience-by-proximity label*: Nearer targets are more salient in being more easily detectable and discriminable and having larger areas of cortical representation. The proximity of targets in conjunction with their SRT has been previously used as a measure of salience.^89^ Similarly, larger targets are more detectable and can also be considered more salient, activating larger neuronal populations at a given eccentricity. To avoid mixing these two types of salience, and to make the experiment easier for participants, Experiment 3 consisted of trials with two equal ring sizes in competition. Here, a salience label is a proxy for proximity, despite this being a great simplification of the saliency literature.^29^ Hence, we use‘ salience-by-proximity’ labels throughout. For trials with competing eccentricity/diameter ratios < 1, the nearer ‘salience-by-proximity model choice’ is opposite to the ‘priority model choice’ (Figure 6A). For trials where the competing ratio is >1 (Figure 6B), the “pure salience-by-proximity model” would also be different from the “pure priority model” in strongly predicting the nearer target.

*Priority-Index:* By modelling the decision signal, we can make explicit choice probability predictions from the empirical SRT data for the Priority Model by ‘racing’ the two choices against each other. Because of natural variability in decision rates between the two choices, the preferred priority choice in Figure 6A falls below 100% towards 50%. Because of this decision noise (as well as the overlap in the CP1 metric in Figure 6B conditions for Salience-by-proximity versus Priority predictions), we computed a ‘Priority-Index’, representing the proportion of trials for which the choices entirely match the Priority model, given the SRT rate variability. Hence, an index of 1 means an individual’s free choice probability was entirely predictable from their SRT distributions. Indices were computed separately for each individual and condition. Because some individuals had directional preferences towards rightward targets, a second set of Priority-Indices were calculated that factored out these biases (see supplemental information and Figures S8 and S9). Both sets of indices gave similar patterns (Figure S9), and the bias-compensated index is presented in Figure 7C.

#### Modeling

##### Mechanistic decision signals

SRT distributions are skewed, but 1/SRT tends to be normally distributed. Thus, plotting reciprocal latency distributions on a probability ordinate scale (e.g. probit or cumulative z-score, Figure S5) yields straight lines (‘reciprobit’ plots^9^). By assuming an accumulating decision variable signal rising to a choice threshold, one can infer the average rate and rate variability of the underlying signal either via a ‘LATER’ or diffusion model^8^^,^^32^ from the SRT distributions. If one aggregates all the decision signal in a single-stage process, increases in the mean decision rate appear as parallel shifts to the left on reciprobit plots (e.g. Figure S5).

*Hick’s law:* Increases in the distance-to-threshold (boundary) has been a common explanation for Hick’s law^90^ in which increasing the number of alternative choices increases response time. The rationale is that the prior probability of any given choice is reduced, lowering the starting point of the decision signal accumulation. On reciprobit axes, this Is seen in an anti-clockwise ‘swivel’ around its intercept to steeper slopes (Figure S7A, top). A pure delay before the accumulation starts also presents as a steeper reciprobit slope, but rotating around a point between the median SRT and the reciprobit intercept (Figure S7A, middle).

*Dual-stage model:* One can improve these descriptive models of SRT by splitting the decision signal into two stages, the first more sensory and driven by a diffusion process, and the second more related to motor preparation.^8^^,^^42^ A schematic of this is shown in Figure 2A. Because our experiments use simple, suprathreshold contrast targets, we assume that the first stage is completed in an approximately constant window of 80 ms, similar to neurophysiological^39^ and human evidence.^40^ Whether one chooses an 80 ms or 100 ms window for this first stage, simply changes the inferred accumulation rate modestly of the second-stage (see ‘pure delay’ above).

##### Explanatory payoff-time model

The descriptive LATER model^9^ became explanatory via application of Bayesian arguments and empirical evidence of SRT modulation with target location likelihood ratio.^6^ The LATER sequential sampling accumulation model is framed as a sequential probability ratio test, which is the minimum-time decision rule for a given level of accuracy under uncertain evidence between two alternative hypotheses.^82^^,^^83^ Despite the myriad successes of the LATER model, Carpenter acknowledged the remaining enigma of the extent of procrastination and variability and even suggested that noise might be deliberately injected to encourage exploratory behavior.^134^

The payoff-time model introduced in the results essentially embodies an exploitation/ exploration trade-off. However, this is a trade-off between exploiting information accumulation from peripheral preview (without the disruption of a potentially unnecessary or unhelpful saccade) and committing to exploring the same region of space with a saccade. Linear increases in precision with time at a given eccentricity were estimated from visual resolution measures.

In the foveola, there is thought to be a one-to-one correspondence between each cone and midget ganglion cell and thus the cone spacing should give a good estimate of the maximal resolution output from the foveola region. We took reciprocal cone spacings from human anatomical data^85^ and fitted these as a function of eccentricity, following conversions from densities to separations based on triangular packing^158^ and on 0.28micron/°.^159^ Nasal- temporal asymmetries in cone spacing are not pronounced within the central 10° of the retina, but we fitted each direction separately with a weighted nonlinear regression (*Temporal = 151.7/sqrt(1+3.19∗eccentricity*); *Nasal=148.7/sqrt(1+3.27∗eccentricity)).* These were similar to previous fits of the anatomical data by Marcos and colleagues, which compared well to their imaging data of the retina *in vivo*.^160^ These resolution functions were used to predict the SRT in Figure 3 from the Payoff-time formula, *Payoff-time = Saccade Duration / (1-r*_*e*_*/r*_*0*_*)*.

At eccentricities beyond the central ∼2°, the ratio of cones to retinal ganglion cells changes. The one-to-one connectivity between the most central cones and ganglion cells is lost, with multiple cones connecting to each ganglion cell at larger eccentricities and so the photoreceptor output is averaged across some region. Thus, the functional decrease in acuity vision exemplified by the cone-only cost function above underestimates the decrease in acuity vision at eccentricities >2°, and hence underestimates the benefit of making saccades to more peripheral targets and would tend to predict longer latencies. Hence, beyond the foveola, we needed a different approximation for the output resolution than that provided by cone spacings. We chose the commonly used resolution estimate of Rovamo and Virsu^87^ (1979; averaged across directions and normalised to *1/(1+0.365∗eccentricity)*). This function was used for the large targets in Experiment 1 (4, 8 and 12° diameter) to predict the SRTs in Figure 4, and in Figure S6 for larger eccentricities.

Payoff-time is dependent on saccade duration (Figure 2). The stereotyped relationship between amplitude and duration of saccades (‘the main sequence’) is quasi-linear for amplitudes >4° and shows a compressive non-linearity at smaller amplitudes.^109^^,^^129^^,^^161^ Small saccade durations in our data were best described by a power law as shown by linear relationships on log-log axes, consistent with previous studies that showed power law fits to be superior to linear ones at small amplitudes.^76^^,^^162^ Saccade duration (T) functions with saccade amplitude (A) were fit for each experiment. There was little difference between leftward and rightward saccades throughout and main sequences were computed in Experiment 1 collapsed across direction. In the monocular recordings of Experiment 2, separate regressions were performed for leftward (nasal) and rightward (temporal) saccades; for amplitudes <4° these gave *T*_*L*_*=10*^*1.22*^*A*^*0.33*^, and *T*_*R*_*=10*^*1.21*^*A*^*0.25*^ (Figure 3).

The one free parameter in the foveolar payoff-time model was a scaling constant. This simply shifted the SRT-Eccentricity function vertically to align with the baseline SRT, which is easily influenced by luminance and contrast contexts of a specific experimental setup. Hence, the choice of 80 ms as a fixed sensory detection and selection time, although consistent with available evidence, is arbitrary from a modelling perspective. Longer Stage-1 latencies are just offset by a smaller constant of proportionality. Function optimization was performed in the Matlab Optimization Toolbox.

The single free parameter model was used to fit ring stimuli data from previously published experiments with embedded attention task,^32^ whose instruction emphasised performance accuracy rather than saccade reaction (black data in Figure 5E).

A second free parameter was added to capture task instructions that emphasise following the stepping target as quickly as possible. Reaction times naturally vary with task instruction. Urgency to react can terminate a decision before the usual decision threshold (‘bound’) has been reached. This termination, or ‘deadline process’, is usually modelled either by a ‘collapsing bound’ or an accelerated decision signal.^144^ Because we previously argued for urgency acting on the accumulation rate, we implemented this ‘deadline process’ free parameter via an acceleration of the decision accumulation rate over the duration of a trial. This second free parameter modestly improved the goodness of fits and is used for the main Figure 1 data.

### Quantification and statistical analysis

#### Eye movement analyses

Offline eye movement records were analysed via a custom-written (Matlab) automated algorithm with graphical interface, which allowed all trials to be manually checked and corrected, if necessary. Eye velocity was estimated by a central difference algorithm, with separation difference matched to minimize the instrument noise of the different eye trackers.^132^ Saccades were identified when the velocity exceeded 15°/s for at least two consecutive samples, and start and endpoints were marked when this velocity fell below a 10°/s threshold. Only the first saccade in a trial was measured. Trials containing blinks during fixation before the first saccade, or during it, were discarded. Trials with saccade latencies <80 ms or >600 ms were excluded for being either anticipatory or potentially unconnected to the visual stimulus. Overall, 93% of trials were included in the analyses.

#### Statistical analyses

Analyses were performed in Matlab Statistics, Curve Fitting, and Optimization Toolboxes. Statistics are presented in the Results section, rather than in Figure captions. Goodness of fit measures were computed for power law fits to empirical data and Nelder-Mead nonlinear minimization fitting of the model equations. An alpha significance level of 0.05 was used. Parametric assumptions were tested. Nonparametric SRT measures were dealt with for individual trials by taking the reciprocal of SRT (promptness) and by taking the median SRT for individual participant’s SRT in each condition. Repeated-measures ANOVA was performed to compare the effects of SRT across group conditions for eccentricity and object size factors. Linear mixed models were used for unbalanced design conditions. Error bars are SEMs in Experiment 1 data, and 95% confidence intervals elsewhere; binomial confidence limits were used for choice data.
