# Supplementary material for: Proximal procrastination of saccades: “paradoxical” delays to larger objects explained by a simple payoff-time heuristic
Source: iScience. 2025 Dec 11;29(1):114426. doi: 10.1016/j.isci.2025.114426 (PMC12818311; doi:10.1016/j.isci.2025.114426)
Supplement: Document S1. Figures S1–S10 [file mmc1.pdf]

iScience, Volume *29*

Supplemental information

Proximal procrastination of saccades: “paradoxical” delays to larger objects explained by a simple payoff-time heuristic

Mark R. Harwood

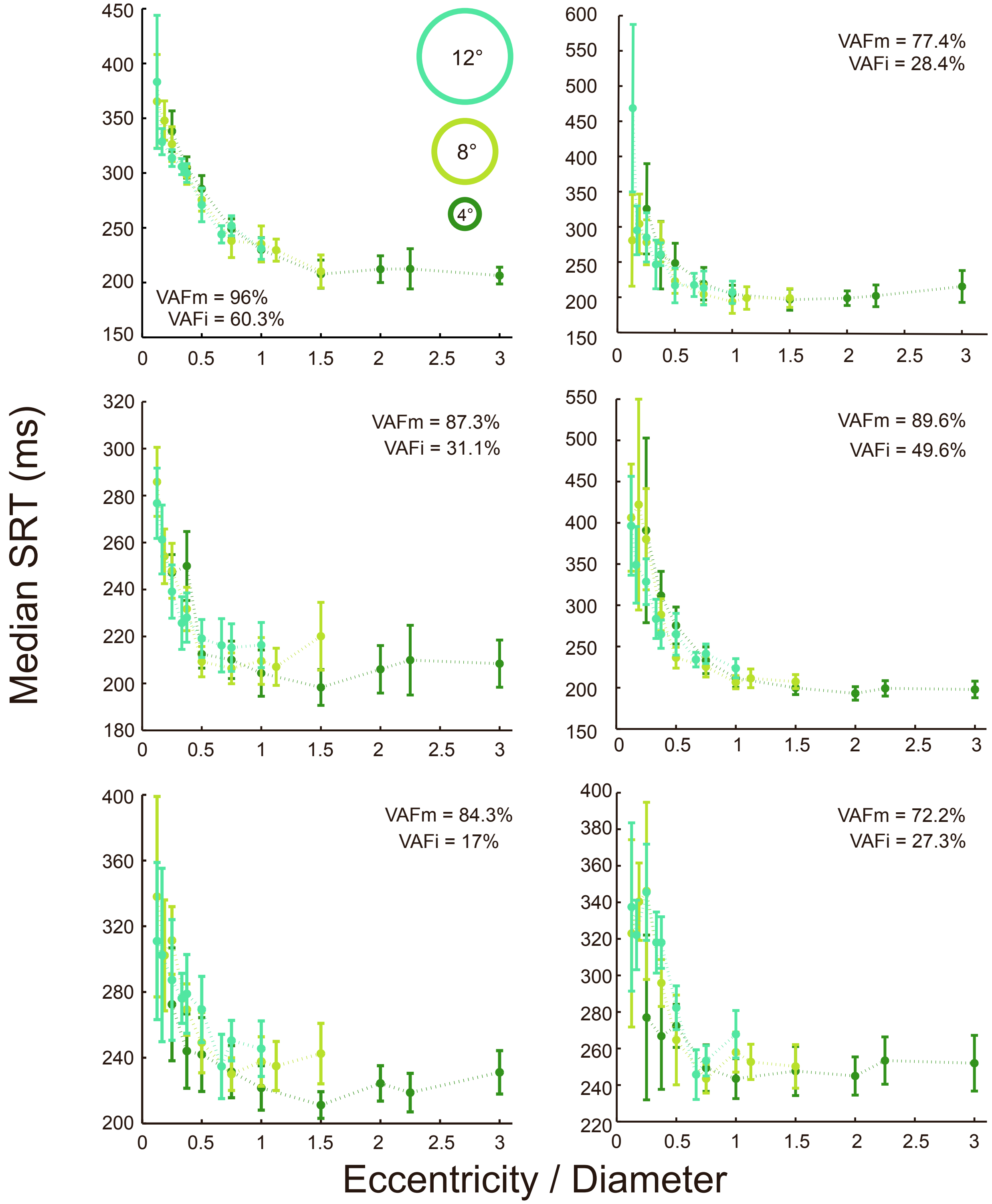

Figure S1. Median SRT for each Individual from Experiment 1, related to Figure 1.

Power law fits of the median SRT over the eccentricity/diameter ratios <= 1 range for each individual gave the variances-accounted-for as stated in the x-axis label (VAFm). Performing equivalent fitting for SRT from each trial in each individual gave the variance accounted for in the top right of each panel (VAFi). Please see STAR Methods for further details. All fits gave significant slopes ($p < .001$). Error bars are 95% confidence intervals.

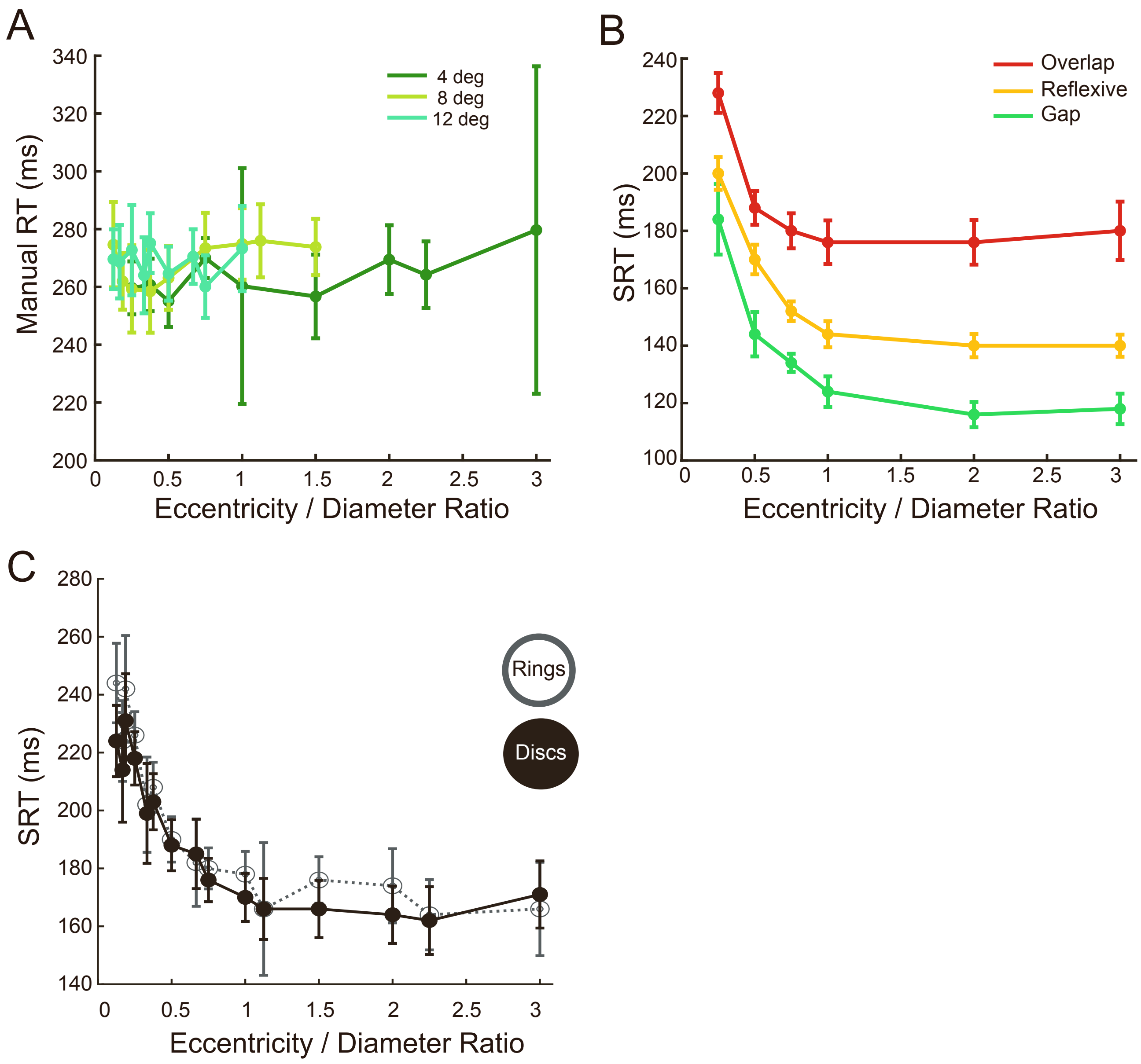

Figure S2. Control experiments from Experiment 1, related to Figure 1.

A) Manual reaction time button presses: the task requested central fixation and responding with a single key press as fast possible on detection of the peripheral target step. The same fixation constraints were followed as in Experiment 1, meaning that if saccades were made to the target more than 80 ms after it stepped, these trials were not excluded. All target steps were equally detectable. **B)** Fixation offset conditions: Median SRT vs eccentricity/diameter ratio in Gap (green), Reflexive (amber) and Overlap (red) paradigms (see Methods for details). **C)** Varied foveal visual stimulation: Median SRT are plotted in response to stepping solid red disc stimuli (full foveal stimulation) of 4, 8, or 12° (solid black symbol), randomly interleaved with thin rings of the same diameter but no stimulus on the fovea (grey symbols). All error bars indicate 95% confidence intervals.

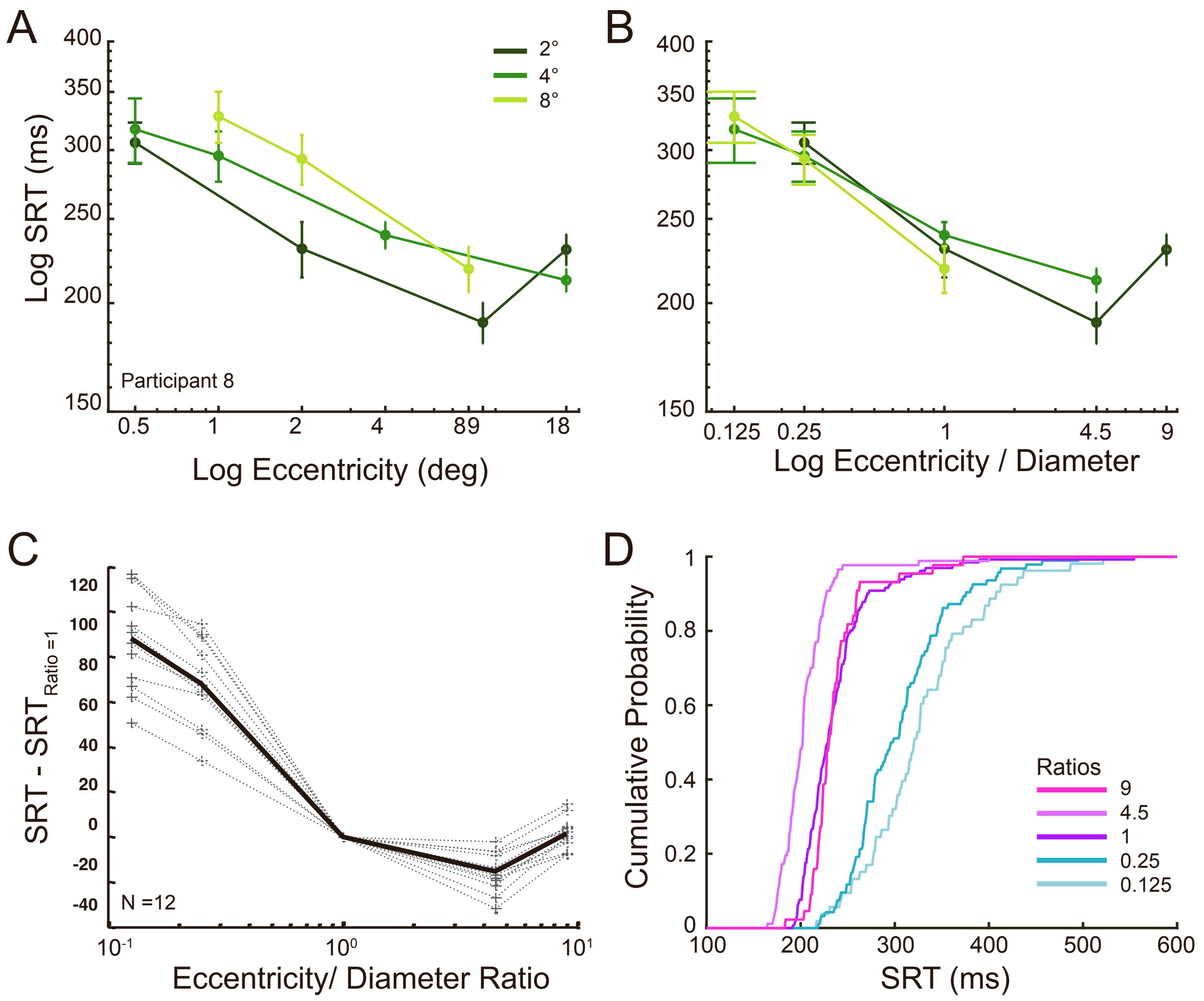

Figure S3. Median saccade reaction times to steps of single ring targets (Experiment 3), related to Figure 1.

A) Single rings stepped from screen centre by +/- 0.5-18°. Ring diameters of 2, 4 or 8° were interleaved randomly across trials. Median SRT vs eccentricity for 2/4/8° ring diameters is plotted for a typical subject. Note the logarithmic scales on both axes and that only those eccentricities tested in the experiment are labelled. Error bars are 95% confidence intervals. B) Median SRT vs Step/Ring ratio (subject from A). C) SRT relative to the SRT at Step/Ring ratio = 1, for each subject (thin curves) and average (bold). Almost all saccade experiments have been in a region beyond our data range, where latencies have largely plateaued (e.g. a typical spot target of 0.3° width, stepping 3° is a ratio of 10). D) Cumulative distribution functions for this Participant 8 showing almost complete separation between ratios>=1 ratios <1.

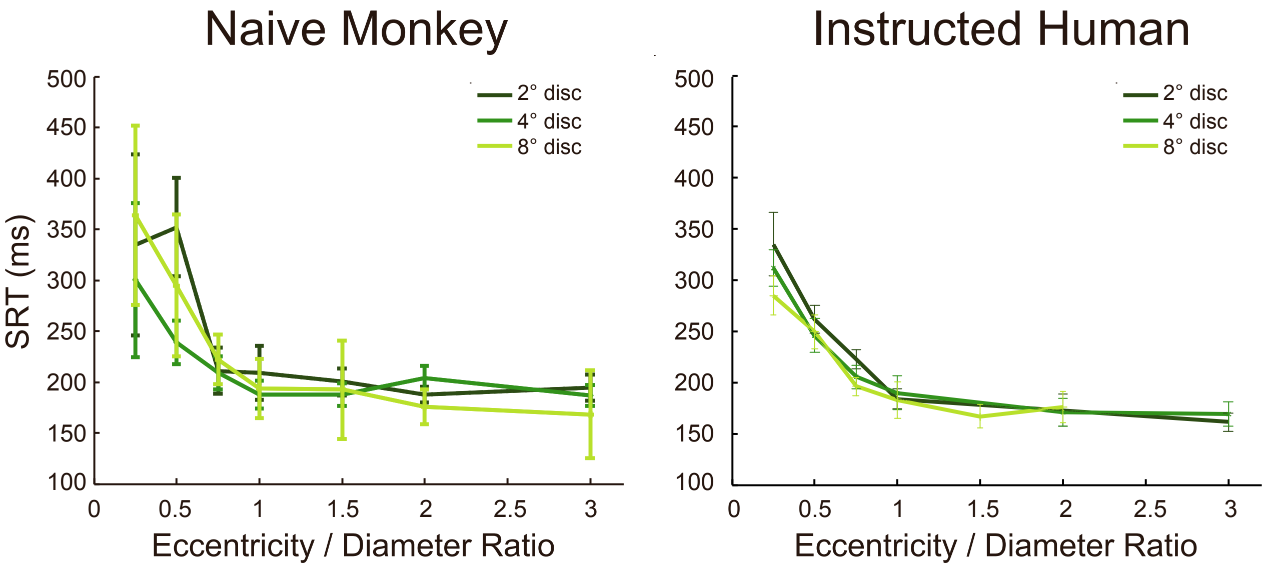

Figure S4. Naive monkey versus instructed human participant, related to Figure 1.

This monkey behavioral data is from the first training session on the spatially extended target task. This data was not included in the companion (Caziot et al., 2025)[60], and permission for its use here has been granted. Median SRT are plotted in each condition. Error bars represent 95% confidence intervals. Note also that discs were used instead of rings, while rings were used in the full study (Caziot et al., 2025).

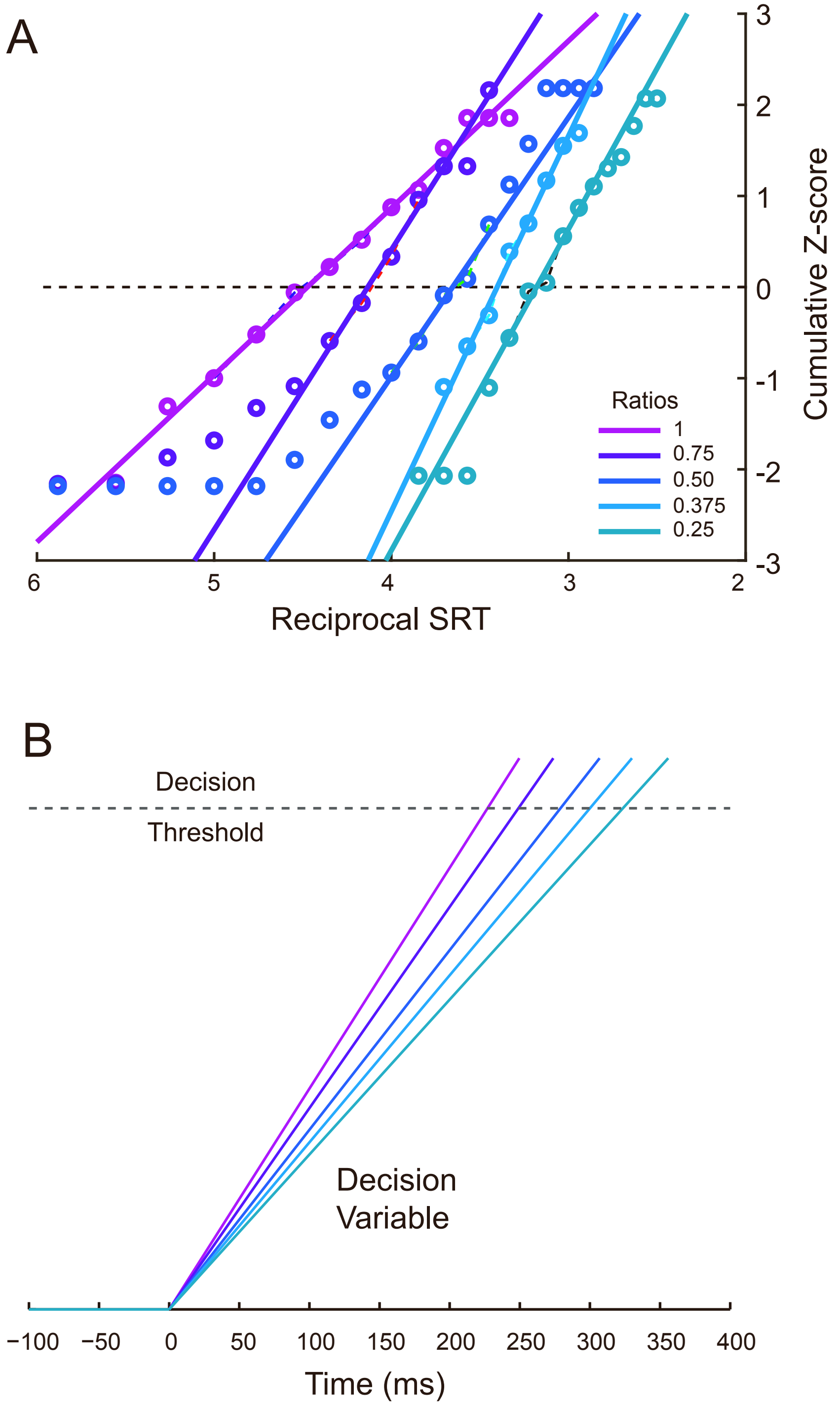

Figure S5. Distribution 'Reciprobit' plots indicating a decision rate change in the underlying accumulation signal, related to Figures 1 and 2.

Top, replotting the cumulative distribution data of Figure 1E in reciprical-probit plots (Participant 1). The mostly parallel shifting curves imply that SRT is shortened predominantly by a change in rate of the underlying decision accumulator (bottom panel- schematic only rather than from the same data). For further elaboration, please see STAR Methods. Larger datasets in humans (Harwood et al., 2008)[32] and monkeys (Caziot et al., 2025)[60] confirm that rates increase with eccentricity/diameter ratio.

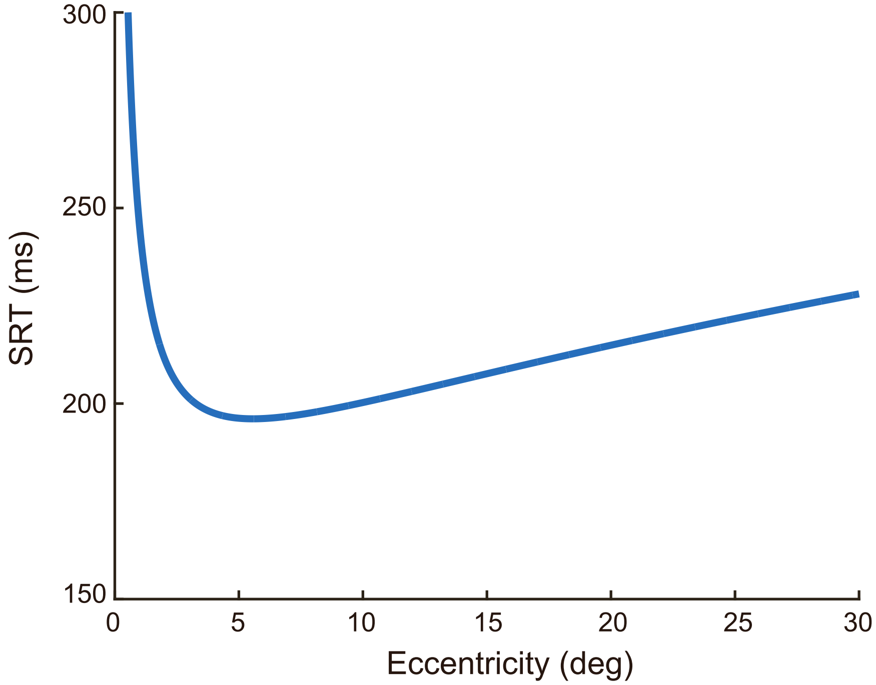

Figure S6. Extension of the visual resolution payoff model beyond the macula for point targets, related to Figure 3.

Payoff-time increases alongside saccade duration for larger movements. In the model, this leads to increasing SRT beyond 10°, forming the “bowl-shape” reported in previous experimental studies (e.g. Kalesnykas and Hallett, 1994)[53]. Note, this is using the resolution function for larger eccentricities and not the cone model shown for within the foveola in Figure 3 (see STAR Methods). There is no adjustment to the fixed stage-1 diffusion detection process.

Visual detection delays are also expected to rise with increasing eccentricity, at least for small objects. Experiments in humans designed to counter these visual delays, by scaling targets according to cortical magnification factors, have found almost identical rises with eccentricity in scaled and unscaled targets (Yates & Stafford, 2011)[49], implying that detection is minimally important in the shape of the eccentricity curve. They used Gaussian targets with a minimum of 2.56° diameter scaled to ~16° diameter over their 5-40° eccentricity range. Conversely, a recent study using point targets (0.1° targets scaled to a maximum of 0.38° over their 2-10°) in monkeys found that this scaling did tend to flatten the eccentricity curve (Zhang and Fries, 2025)[50], implying that this is due to changes in the visual detection Stage-1 process (see Figure 2A).

Given the more complex and uncertain nature of SRT functions at higher eccentricities, beyond our recorded data range, we caution that this extended-eccentricity model is for illustrative purposes only. Zhang and Fries (2025) argued that pre-saccadic attention shifts increase with eccentricity, and these covert shifts explain the increasing SRT-eccentricity function. Note, that their required covert attention shift duration increases are comparable with the saccade duration increases responsible for the rise in the above figure.

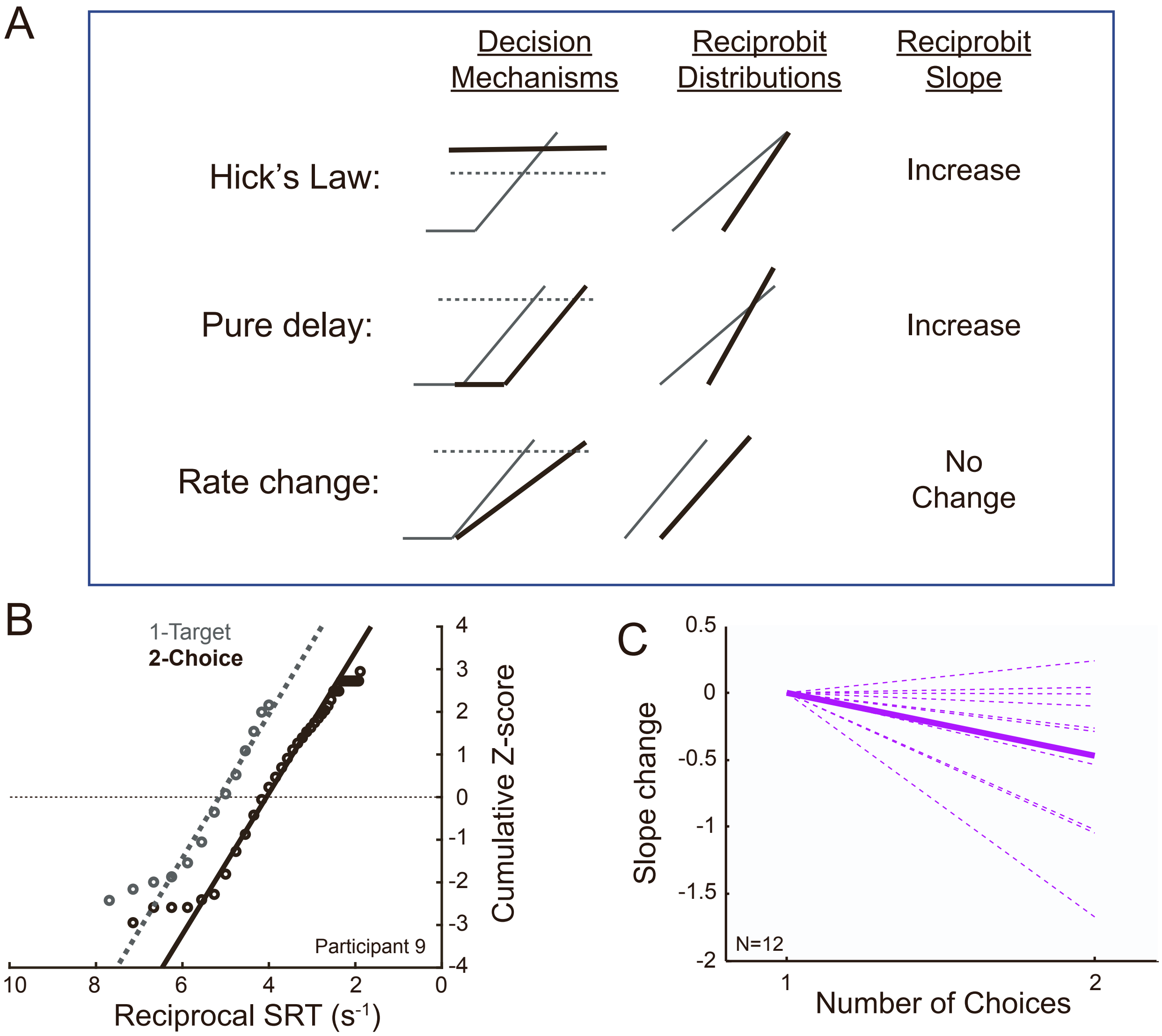

Figure S7. SRT increases with two choices entail mutual inhibition of decision rate, rather than a Hick's Law change in distance-to-threshold, related to Fig. 7A.

(A) Three decision mechanisms for the slowed SRT in a 2-AFC condition (left). and consequences for saccade distributions on reciprobit scales (middle and right). Top of panel A, increased threshold distance (Hick's Law; thick line) causes an anti-clockwise swivel in the SRT distribution; Middle, a pure delay (e.g. from longer visual processing) before decision accumulation begins leads to reciprobit curves crossing at a point above median SRT; Bottom, reduced decision rate causes a parallel distribution shift.

(B) Representative participant comparison of 1-AFC single-ring control condition (gray dotted curve) and 2-AFC ring free choice (black solid curve) for eccentricity/diameter of 1 condition. SRT is shifted in parallel to longer SRT (shorter reciprocal latency, 'promptness'). Slopes were fit to the central 95% of the distributions.

(C) The 12 participants' reciprobit slope changes (dashed lines) and group average (solid) are shown in the most frequent eccentricity/diameter of 1 condition. Both Hick's Law and pure delay predict increasing reciprobit slopes, compared to a no-change in slope for a pure rate change. The data are consistent to a mutual inhibition rate decrease in the two-choice condition, with a small threshold *decrease*, because the slopes were significantly decreasing (mean slope change = -0.43, $t(11)$, $p = .02$, $d = 0.77$). In single-choice conditions, a similar threshold decrease has been found at weaker saccadic intensities (lower eccentricity/diameter ratios) in human data[32] and the neural correlates in the companion monkey paper[60].

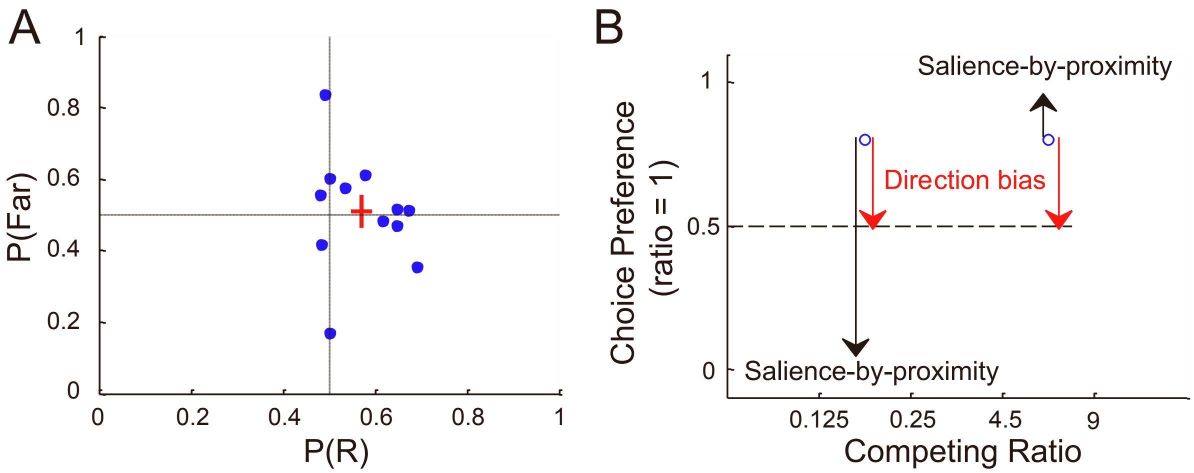

Figure S8. Directional and Proximity biases in the 'Free Choice' Experiment 3, related to Figure 7.

(A) Probability of choosing the more distant (P(Far)) or the right target (P(R)) is shown for individuals (blue points) and group 95% confidence intervals (red cross). There was no overall bias in proximity (the ratio = 1 target was either the nearer or farther target on half the trials). Rightward probability was biased overall due to 5 biased participants.

(B) Effect of salience-by-proximity and direction bias on the preference of choosing the ratio of one target. Circle shows a choice preference predicted purely from the Priority race model. Directional biases, which are unbiased with respect to ratio, will push CP(ratio =1) towards 50% (red arrows). Salience-by-proximity biases will bias choice in favour of nearer targets and, thus, are shown as pushing the CP(ratio of 1) towards 0 for competing targets at ratios <1, and

In comparing the simulated predictions of a pure priority-model to the empirical choice preferences, we first considered how directional bias unrelated to stimulus features would deviate the preference data.

Nearly half our subjects exhibited statistically significant biases towards making rightward choices, which were independent of ratio condition, proximity of target (trial Type) or SRT (Fig. S8A). Because the directional bias could not be explained by stimulus properties or SRT, we suggest it arises from idiosyncratic top-down biases, which could be modelled by assuming that this process ultimately determines rightward choices on a proportion, R, of trials (leaving 1-R trials determined by stimulus or SRT race models that are unbiased with respect to direction).

The probability of choosing right then is: $CP_R = R + 0.5*(1-R)$. Hence, an observed 60% preference for rightward choices is assumed to arise from 80% trials based on directionally unbiased sensorimotor factors and 20% of biased trials (R = 0.2). Similarly, there is no reason for the R trials to be biased with respect to our main metric, $CP_{empirical}$, the choice preference to the ratio of one target step. Hence, $CP_{empirical} = 0.5*R + (1-R)*(\text{“real” } CP_{stimulus\ driven})$. This unbiased effect of R tends to push the empirical choice preference for ratio of one targets towards 0.5 (Fig. S8B, red arrows). A similar argument has been used previously to account for conscious guessing corrupting the choice preferences of an underlying stimulus driven process (Leach and Carpenter, 2001)[91].

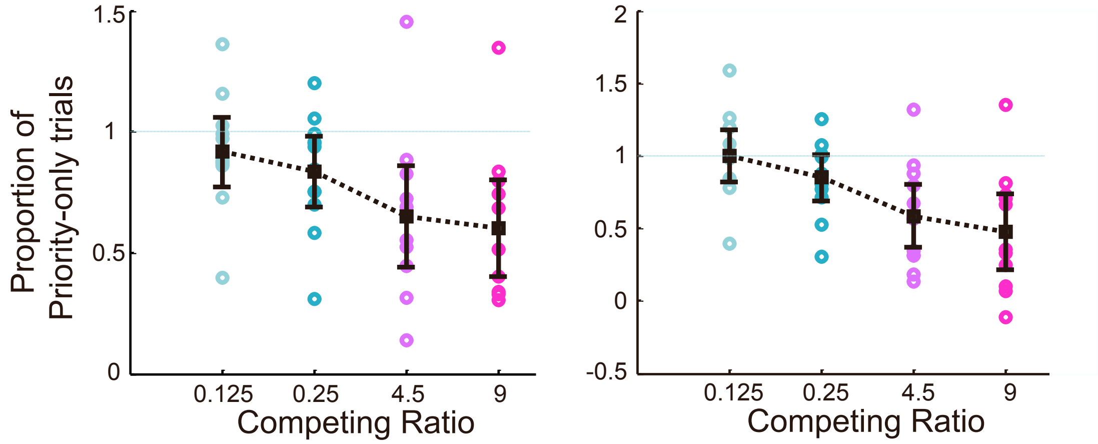

Figure S9. Proportion of Priority-only trials in Experiment 3, related to FIgure 7.

Proportion of Priority-only trials (Q) against ratio of competing target. Left panel shows Q without correcting for directional biases, while the right panel includes directional bias corrections. Individual data and group averages with 95% confidence intervals (black) are shown. Note at lowest ratio, Q=1, indicating that salience-by-proximity is not required to explain the data in this condition and design. The right panel forms the ‘Priority Index’ of Figure 7C.

We followed a similar logic to that under Figure S8 to separate out the priority and salience factors of $CP_{stimulus\ driven}$ (in part, to obviate the need for a more explicit model of salience effects). We first assumed that the “real” $CP_{stimulus\ driven}$ was determined from our explicit model of reaction times and priority, but that on a certain proportion of trials (1-Q) the effect of visual salience ultimately determined the choice. On the majority of trials, Q, priority determined the outcome according to the race model prediction of $CP_{priority\ race}$.

We assumed that salience will bias this choice towards the nearer target, hence reducing CP(ratio = 1) when the competing target is at ratio <1, and towards 1 when the competing target is at a ratio >1 (Fig. S8B, black arrows). We can express this as: $CP_{stimulus\ driven} = Q^* CP_{priority\ race} + S^*(1-Q)$

with S = 0 for competing ratios <1, and S = 1 for those >1.

Combining equations:

$$CP_{empirical} = 0.5^*R + (1-R)^*(Q^* CP_{priority\ race} + S^*(1-Q)).$$

In the Results section, we apply these formulae to compensate for directional biases and to compute the proportion of priority-only trials, Q, at each ratio condition. This provides a metric for assessing the goodness-of-fit of our Priority model, without the necessity to apportion specific salience effects on rate or rate variability of an accumulating decision variable. That is, Q = 1 would indicate that the Priority race model is sufficient to explain all the observed choice preference without any need for salience.

The corresponding values of Q before taking into account the rightward bias, R, were: 92, 84, 65, 60% for ratios 0.125, 0.25, 4.5, 9, respectively (Fig. S8C, left). Taking R into account, increased the measure of Priority importance at the lowest ratio, but had limited influence in other conditions (Priority Indices: 100, 85, 58, 48%). The latter are those presented in Figure 7C.

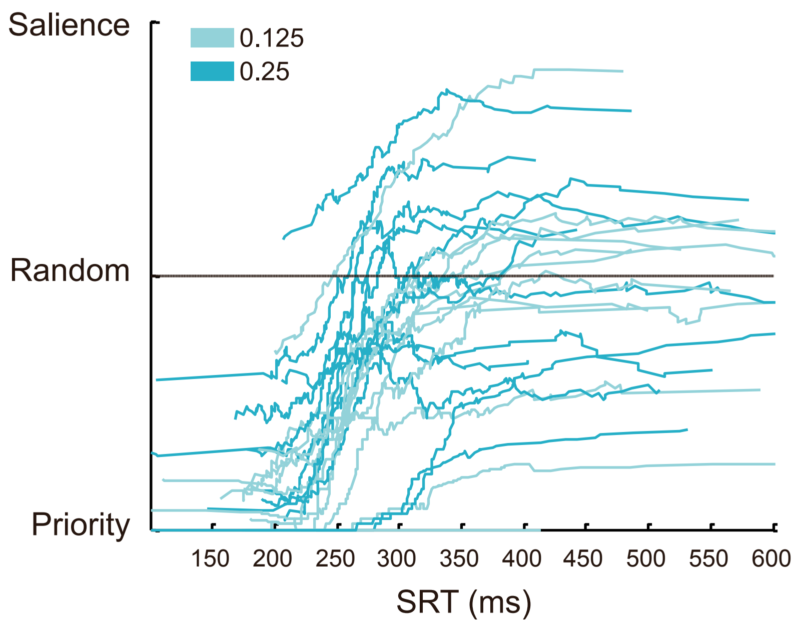

Figure S10. Choice Probabilities as a function of SRT for each individual at the two lowest ratio conditions ('Type 1' trials), related to Figure 7.

Locally-weighted regression curves (LOWESS) show that even subjects with weaker preferences towards choosing the ratio = 1 targets (''Priority' ordinate label above) have strong preferences for choosing the ratio = 1 targets at SRTs in the range 150-250 ms. These curves through the individual data are shown as 95% confidence bands in Figure 7D.
